# Compliant Polymeric Sheet‐Based Heat Exchangers

**DOI:** 10.1002/advs.202520009

**Published:** 2026-02-21

**Authors:** Richard J. Fontenot, Loic Duggal, Sofia Urbina, Barclay Jumet, Anoop Rajappan, Daniel J. Preston

**Affiliations:** ^1^ Department of Mechanical Engineering Rice University Houston USA; ^2^ Department of Physics and Engineering Physics Tulane University New Orleans USA; ^3^ Rice Advanced Materials Institute Rice University Houston USA; ^4^ Rice Space Institute Rice University Houston USA; ^5^ Carbon Hub Rice University Houston USA

**Keywords:** compliant, deployable, sheet lamination, soft materials, thermal management

## Abstract

Heat exchangers provide essential thermal management, spanning from the food industry to chemical processing and beyond; however, they are often made from metals with correspondingly high material and manufacturing costs, along with susceptibility to fouling and corrosion. To address these limitations, researchers have worked to achieve similar performance with heat exchangers made from polymers, but early designs remain expensive, geometrically complex, and limited by low thermal conductivities and operational temperatures. Additionally, previous studies are primarily empirical in nature and lack a sufficient link to theory to enable future design. In this work, we create and characterize heat exchangers made of thin (∼50 µm), transparent polymeric sheets that exhibit heat transfer coefficients up to 2000 W/m^2^K while providing a heat exchange capacity per cost 2 to 4 times greater than metal and previous polymer counterparts. These heat exchangers are deployable up to 60 times their initial volume, allowing for compact storage and use in volume‐constrained applications (e.g., takeoff preceding space missions or terrestrial shipping logistics), and their performance can be predicted accurately by an analytical model. This sheet‐based approach enables effective heat exchange using polymeric materials while also providing in‐situ flow visualization, device‐level deployability, and flexibility for compact thermal management.

## Graphical abstract

This work presents flexible, transparent, and deployable polymer heat exchangers that achieve a heat transfer capacity per cost up to 4x higher than the state of the art, surpassing both metal and other polymer counterparts. The tube‐on‐tube design enables efficient heat transfer without flow occlusion, while the lightweight, corrosion‐resistant, and expandable architecture supports diverse thermal management applications.

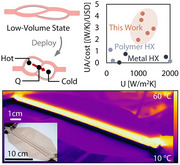

## 1 Introduction

Our world faces increasingly enormous energy needs, with an estimated 180000 terawatt hours of energy consumed per annum [1]. More than half of this total energy consumption involves the transport, generation, or usage of heat at the point of consumption in some manner [2]. In these use cases, the exchange of heat between distinct fluids is a cornerstone of energy systems, from vehicle radiator cooling to solar thermal systems, and even extending to general waste heat recovery. Heat exchangers played an important role in industrialization in the past, and they continue to play a central role in meeting our energy needs in the 21st century [3].

Metal‐based heat exchangers are prevalent in industry and in the academic literature, primarily due to their high material‐level thermal conductivity and mechanical strength [4]. However, issues with these metal‐based heat exchangers are well documented, including (i) high material costs, (ii) complexity of fabrication, and (iii) fouling and corrosion concerns [5]. These challenges have become seminal problems in the world of thermal management, especially in fields such as desalination that rely on heat exchangers in highly corrosive environments for long‐term usage [6, 7]. Historically rooted assumptions appear to have maintained a focus on metal‐based heat exchangers and perhaps prevented alternative material choices from widespread adoption in industry, but alternatives to metals hold great promise, with several important examples found in nature [8]. For instance, certain species such as lamnid sharks and tuna contain bundles of blood vessels that act as counterflow heat exchangers in order to manage body temperature [9]. In these networks of blood vessels, called the *rete mirabile*, higher temperature blood from the heart warms cooler blood that was exposed to a cold environment before it returns to the heart. In this instance, nature demonstrates that effective heat exchange can occur across low thermal conductivity blood vessels (nearly three orders of magnitude less thermally conductive than metals), establishing that alternatives to metal are viable and have precedent in biological systems [10].

For traditional thermal applications, compact heat exchangers made from low thermal conductivity materials have been reported in the literature as early as 1965, with researchers seeking the known benefits of polymeric materials, including their low cost, light weight, and fouling‐ and corrosion‐resistant characteristics compared to metals [11, 12, 13, 14, 15, 16]. 3D‐printed heat exchangers in particular have seen a substantial increase in research over the past decade, as researchers seek more design customization and optimization than past techniques [17]. Innovation via polymeric 3D‐printed heat exchangers provides the benefits of a tailored geometry while remaining compact, but often comes with higher pressure drops and a complex and time‐consuming fabrication process [18]. Furthermore, the literature lacks detailed experimental analyses of 3D‐printed polymer heat exchangers supported by analytical modeling to enable tailorability and optimization. Additionally, cost considerations are typically glossed over, and reported polymer‐based solutions do not provide a clear cost advantage over metal counterparts, although some manufacturers have started to produce off‐the‐shelf polymeric heat exchangers, mostly through extrusion‐based fabrication methods and with limited characterization [8]. Overall, while polymer heat exchangers offer great promise in terms of performance and customization, they still require further exploration to address issues such as high pressure drops, complex fabrication, limited maximum temperatures, and a lack of detailed cost analyses [18, 19].

**FIGURE 1:**
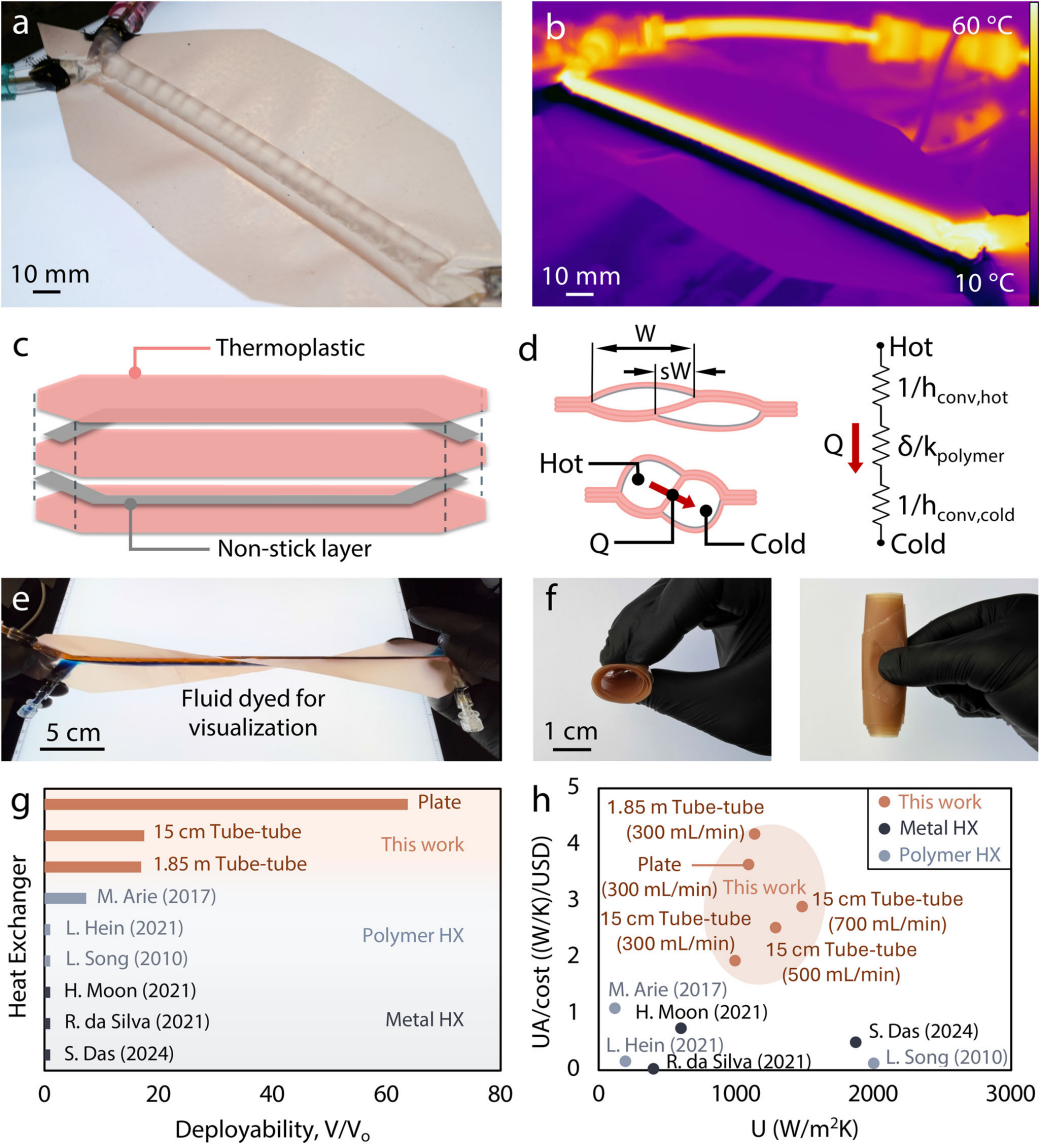
Sheet‐based polymer heat exchangers shown in (a) the visible and (b) the infrared (IR) spectrum showing the separated two‐fluid flow consisting of a hot (60°C) and a cold (10°C) stream for a Δ*T* of 50°C. (c) Exploded view of the sheet‐based devices with the non‐stick layer delineating the channels to be filled with fluid. (d) Cross‐sectional view of an unactuated (top) and deployed (bottom) sheet‐based heat exchanger, with the relevant thermal resistance network. (e) Demonstration of flexibility while filled with two separate dyed fluids. (f) Demonstration of flexibility while unfilled, demonstrating deployability. (g) Comparison of this work to other polymer and metal heat exchangers regarding the deployability ratio (Section S8), comparing the devices to their initial volume prior to operational usage. (h) The overall heat transfer coefficient and the heat transfer capacity normalized by cost compared to other heat exchangers (cost matrix in Section S3) for an experimental flow rate range of 300 mL/min to 700 mL/min.

In this work, we capitalize on the known benefits of polymer heat exchangers to create a class of low‐cost and scalable heat exchangers that are flexible and transparent and can be deployed from an initially thin (∼250 µm) state (Figure 1). We first introduce and characterize a heat exchanger with a tube‐on‐tube design, where overlapping compliant fluidic channels transfer heat but the overlapped region does not buckle nor occlude flow [20]. This tubular channel design allows for development of an analytical heat transfer model—based on applicable Nusselt number correlations—to predict performance and validate designs for tube‐on‐tube heat exchangers with good accuracy compared to the experimental results. In addition, we demonstrate the scalability of our designs in 3 ways: (i) a serpentine design with ∼2 meters of heat exchange length; (ii) a conventional plate‐and‐frame heat exchanger; and (iii) a single channel tube for immersion heating and cooling. The polymer heat exchangers presented in this work exhibit competitive performance in terms of both an overall heat transfer coefficient comparable to the state of the art (800–2000 W/m^2^K) and a heat transfer capacity per cost of 2–4x the state of the art, considering both metal and other polymer heat exchangers. The deployable architecture of our sheet‐based polymer heat exchangers, with an expansion of up to 60x the undeployed volume, lends itself to volume‐conscious applications like transient computational cooling or pumped fluid loops for satellite thermal control [21]. Furthermore, we demonstrate (i) in‐situ visualization of flow regimes enabled by the transparent architecture of the heat exchangers, (ii) wearable cooling for enhanced performance made possible by their compliant nature enabling contact with the shape of a user's body, and (iii) resistance to acid corrosion based on their polymer‐based construction. In summary, our tube‐on‐tube design, polymer sheet‐based fabrication method, and demonstrations of single‐channel and plate heat exchangers outline a future for the deployment of polymer heat exchangers in scenarios including highly corrosive environments, compact aerospace applications, and thermal management for desalination and other terrestrial applications at the water‐energy‐food nexus.

## 2 Results

### 2.1 Fabrication and Assembly of Sheet‐Based Polymer Heat Exchangers

The fabrication technique presented in this work centers around sheet‐based lamination, in which a thermoplastic material at an elevated applied temperature and pressure (details outlined in Section S1) melts, reflows, and then bonds to another thermoplastic. For materials, we selected nylon and polyethylene as the representative thermoplastics to fabricate our devices. We chose these materials because the sheets are optically transparent, thin (∼50 µm), and cost‐effective (∼4.25$/m^2^) with promise for widespread use. To create the channel for fluid flow and heat exchange, we place a non‐stick masking layer in between the thermoplastic sheets to delineate the channel and form the desired channel geometry (detailed in Section S1). For the heat exchangers, we create two separate channels to house the two separate fluid flows. The archetypical sheet‐based heat exchanger stack‐up in Figure 1c contains three pieces of thermoplastic and two masking layers to define the channels, with two of those thermoplastic layers on the outside and one serving as a separating layer between the two non‐stick layers. Finally, we align the layers to prescribe the overlap fraction *s* for tube‐on‐tube heat exchangers and then laminate the aligned materials with a heat press. After fabricating the device, we attach port fittings with epoxy to the device, resulting in a quick and straightforward fabrication technique (detailed in Section S1). We use the sheet‐lamination technique in this work and highlight it as an effective alternative to the 3D‐printed methodologies that are prevalent in the literature [22, 23, 24, 25, 26]. We characterize the maximum pressure of our system in Section S9 based on an approach established in previous work [27, 28]. The accessible, low‐cost, and scalable stacking technique of polymer sheets in this work contrasts with the complex and rigid 3D‐printed heat exchangers made of either polymers or metals. This design approach deepens the characterization of this manufacturing technique and demonstrates its use‐cases for future applications in thermal management and beyond.

### 2.2 Tube‐on‐Tube Heat Exchanger Characterization

For characterization of our polymer heat exchangers, we begin with the tube‐on‐tube design in which two compliant channels overlap by a prescribed amount to allow for flow in both channels while remaining separated. In our prior work on soft fluidic logic for wearable assistive devices, we investigated the structure of overlapping fluidic channels and observed a buckling instability beyond a 78% overlap of the channels; prior to this instability, at lower overlap fractions, the sheet separating the two channels remains under tension and approximates a plane [20]. We exploit this particular phenomenon prior to the instability occurring for our tube‐on‐tube heat exchanger design; specifically, we begin with the 78% overlap design to maximize the area of heat transfer without buckling the channel walls, and we decrease in overlap fraction to 56% and 34%, respectively, to examine the change in performance in 22% decrements, with this decrement selected based on the difference from 100% to 78%. In addition to studying the effect of the overlap fraction, we investigate various lengths of this design, from compact 10, 15, and 30 cm designs to a scaled‐up serpentine tube‐on‐tube design (∼2 m). Furthermore, we predict the performance of the tube‐on‐tube devices via Nusselt number correlations derived from the literature of flow in a pipe detailed in Section S4. For all experiments, heat losses to the environment are negligible (Section S2) and all results shown are based on temperatures measured from thermocouple data (Section 4).

#### 2.2.1 Tube‐on‐Tube Heat Exchanger Performance Modeling

We utilize a conventional technique referred to as a heat exchanger performance calculation, where we analyze a given heat exchanger's performance to determine the heat transfer rate and outlet stream temperatures for specified conditions of the inlet temperatures and flow rates [29, 30]. For our design case, we measure the temperature at both the inlet and the outlet along with the flow rates of both streams on the inlet side. With this information, we conduct the heat exchanger performance calculation using the log mean temperature difference (*LMTD*) and effectiveness‐NTU methods to determine the overall heat transfer coefficient (*U*) and the rate of heat transfer (*Q*) between two fluid streams (Equation (1)) [31]. One of the most important terms for this study is the overall heat transfer coefficient, i.e., the inverse of thermal resistance, which takes into account the convective resistances of both streams and conductive resistance across the thin layer that separates the two streams; for the modeling approach used in this paper, we define these resistances in Equation (2), yielding *U*_model_. We can calculate *Q* across each stream using the energy balance equation (Equation (S1)) with a known temperature difference, mass flow rate, and heat capacity rate. Taking the average of the heat transfer rates of each stream from Equation (S1), we insert this value into Equation (1) to obtain the overall heat transfer coefficient *U*, or, when including area, the heat exchange capacity, *UA*.

(1) $$Q\;=\;UA\Delta T_{\mathrm{LMTD}}$$

(2) $$U_{\mathrm{model}}=\frac{1}{\sum R}=\frac{1}{\frac{1}{h_{\mathrm{hot}}}+\frac{\delta }{k_{\mathrm{mat}}}+\frac{1}{h_{\mathrm{cold}}}}$$

**FIGURE 2:**
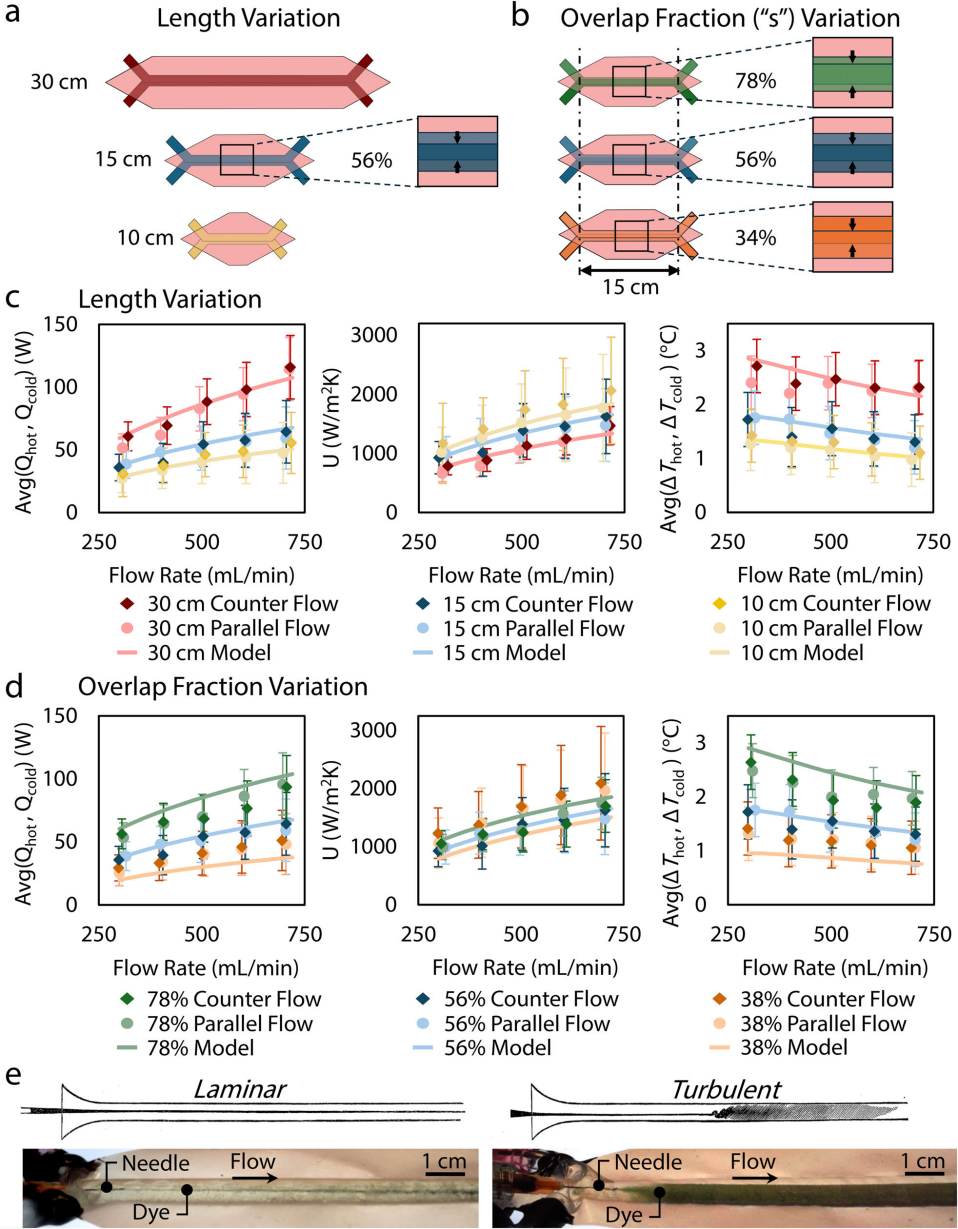
Polymer tube‐on‐tube heat exchanger characterization for a hot inlet (60°C) and a cold inlet (10°C) with a flow rate range of 300 mL/min to 700 mL/min. (a–c) Length variation impact on heat exchanger design performance in terms of average rate of heat transfer (*Q*_avg_), overall heat transfer coefficient (*U*), and average temperature difference between two streams (Δ*T*_stream_) for the lengths of 10 cm, 15 cm, and 30 cm. (b–d) Overlap fraction variation impact on performance in terms of *Q*_avg_, *U*, and Δ*T*_stream_ by for overlap fractions of 34%, 56%, and 78%, with the maximum based on the 78% limit before a buckling instability of *A* as determined from prior work [20]. (e) Photos of flow visualization demonstrating flow profiles with fluid dye visually similar to the quintessential fluid mechanics paper by Osborne Reynolds [38].

In Equations (1) and (2), *A* is the heat exchange area (A = *s∙W*∙*L*_channel_, where *s* is the overlap fraction and *W* is the deflated channel width). *h*_hot_ is the convective heat transfer coefficient in the hot stream, δ is the thickness of the polymer sheet from which the heat exchanger is constructed, *k*_mat_ is the thermal conductivity of the polymer sheet (*k*_nylon_ = 0.25 Wm^−1^K^−1^ for all thermal testing and *k*_polyethylene_ = 0.40 Wm^−1^K^−1^ with further detail in Section S4), and *h*_cold_ is the convective heat transfer coefficient in the hot stream. We employ an analytical model (detailed in Section S4) to predict the performance of tube‐on‐tube heat exchangers. Specifically, utilizing the effectiveness‐NTU method for known inlet conditions but unknown outlet conditions, we can predict the overall heat transfer coefficient *U*, the outlet temperatures to obtain the relevant Δ*T* for each stream, and the rate of heat transfer *Q* between the two fluids. Our primary goal is to obtain the convection coefficients in Equation (2) to predict *U*; however, the Nusselt number must first be found to obtain the convective terms in the equation. We select two correlations for the laminar and turbulent regimes, respectively, and utilize a characteristic length of *A*_xc_^1/2^ instead of the diameter, due to the non‐circular cross‐section of the devices as shown in Figure 1d. For laminar flow, we select a correlation from Muzychka and Yovanovich (Equation (S5)) for non‐circular ducts that presents itself as a versatile approach with a ±15% accuracy in the laminar regime [32, 33, 34]. Next, for transitional and turbulent flow, we elected to use the correlation by Sarmiento et al. (Equation (S6)) built from a Gnielinski correlation, due to its demonstrated accuracy in the transitional and turbulent regimes across the literature [35, 36, 37]. These correlations yield the required Nusselt number for predicting the convection coefficients of the fluid streams, *h*_hot_ and *h*_cold_, with a more detailed explanation on the selection of Nusselt number correlations available in Section S4. We base our models shown in this work on the parallel flow model only, due to the small differences (<0.1% detailed in Section S5) from the counter flow result at these length scales. The models show good agreement with the experimental data in Figure 2c,d, with some deviations that can be attributed to varied cross‐sectional area, short entry regions, and minor flow rate variations within an individual test. We then characterize the tube‐on‐tube designs and model their results in two ways to further explore this tube‐on‐tube design, through varying (i) the overlap fraction and (ii) the heat exchanger length. [Correction added on 24 February 2026 after online publication: equation 1 and 2 are updated in this version.]

#### 2.2.2 Tube‐on‐Tube Size‐Based Characterization

We first characterize the tube‐on‐tube heat exchangers through length variation to analyze the impact device length has on performance. For all experiments, we conduct trials with deionized (DI) water‐water heat exchange with the fluid streams at inlet temperatures of 10°C and 60°C, respectively (Δ*T* = 50°C), while varying the volumetric flow rate from 300 to 700 mL/min to capture performance for a variety of flow regimes including laminar, transitional, and turbulent flow. For the length variation study, we hold the overlapping channel fraction constant at 56% and study compact lengths of 10, 15, and 30 cm to examine the impact of these changes in length. We expect that the increase in the length of the tube‐on‐tube heat exchanger while maintaining a constant channel diameter will increase both *Q* and Δ*T*, respectively, as a greater area of heat transfer is directly proportional to a higher *Q* in Equation (1), which results from a larger Δ*T*. Viewing the experimental results, we do see an increase in *Q* and Δ*T* as the length of the heat exchanger increases. Additionally, the overall heat transfer coefficients (*U*) remain close per tested flow rate despite the length changing in Figure 2a, which follows our expectation because the *U* quantity is per unit area. Nevertheless, the *U* values of the 10 and 15 cm designs are above that of the 30 cm design; this result can be rationalized due to the short entry length of the devices, which results in flow mixing and therefore higher‐than‐expected performance in terms of *U*. We also see an increase in *Q* and *U* with volumetric flow rate, because an increasing mass flow rate is proportional to a larger *Q* and *U* in Equation S1. Lastly, the Δ*T* of all lengths decreases with an increasing flow rate due to the decrease in dwell time (i.e., the time of contact between hot and cold streams). All of these trends are evident for both a counterflow and parallel flow orientation, which were both tested for this study; the process for the uncertainty analyses for all trials is provided in Section S6. The analytical models presented in Figure 2 are models based on parallel flow operation only, due to the very close similarity (< 0.1%) of the parallel to the counterflow model under the conditions tested in this study, leading to one flow model accurately representing the performance for both cases. This similarity in prediction can be attributed to how compact (< 30 cm) the heat exchangers are in this study, with low overall temperature change in each stream relative to the temperature difference from one stream to the other. In summary, we validate that increasing the length of the heat exchangers subsequently increases the performance through experiments and analytical modeling, mostly due to the increase in the area of heat transfer.

#### 2.2.3 Tube‐on‐Tube Overlap Fraction Characterization

Next we vary the tube‐on‐tube overlap fraction. We determine the specific overlap fraction of the two fluidic channels through a process outlined by Rajappan et al., where two sheet‐based fluidic channels are overlapped by a prescribed amount before being pressurized [20]. When the overlap percentage of two channels exceeds 78%, the dividing layer buckles and partially occludes one channel. We note that, upon preliminary testing with a 100% fully overlapped design, we observed flow instabilities in which the dividing layer oscillated between occlusion of the hot and cold streams, a behavior that has the potential to further improve convective heat transfer [39]; this phenomenon warrants additional study in future work. Due to this potential for oscillatory behavior at high overlap fractions potentially impacting operational stability and consistency, we limited the overlap fraction to 78% or less in this work, which informs a potential maximum area of heat transfer for the tube‐on‐tube configuration to exhibit predictable steady operation. We begin testing with the 78% overlap design (maximum *A* before buckling), sequencing down by an overlap decrement of 22% from the maximum overlapped design, resulting in overlap fractions of 78%, 56%, and 34% being considered in this work. For overlap fraction testing, we hold the heat exchanger length constant at 15 cm to isolate the effect of the overlap changing. We predict that an increase in overlap fraction will result in an increase in performance in terms of *Q* and Δ*T*. Our experimental results validate our prediction: the increasing percentage of overlap fraction in Figure 2b increases both *Q* and Δ*T* due to the increase in the area of heat transfer. The results for *U* are within the same range as each other, as expected, but the lower overlap fraction of 34% has marginally higher results relative to the model. This difference may be due to the 34% overlap's cross‐sectional area more closely following a circular shape than the *A*_xc_^1/2^ characteristic length that the model uses, leading to the experimental results deviating above their prediction, all while remaining within the uncertainty bounds of the experimental data. Additionally, the effects of flow orientation for counterflow and parallel flow had a relatively minor difference of approximately 5% across all experiments due to the compactness of these heat exchangers and due to the experimental error outlined in Section S4. We show that the 78% overlap heat exchanger design remains stable during operation, without buckling, and allows for consistent fluid flow to occur for heat exchanger operation.

#### 2.2.4 Flow Visualization and Flow Regime Determination

The thermoplastics we use in this work are transparent in the visible spectrum, contrasting with opaque metals that are conventionally used for heat exchangers. In addition to the efficacy of our sheet‐based heat exchangers in terms of heat transfer performance, this transparency presents opportunities for engineers to visualize the fluid flow inside their heat exchangers. This opportunity allows for a multitude of applications that include operational observation, flow regime determination, and live troubleshooting. Furthermore, outside of their own thermal engineering merits as thermal management devices, these transparent thermoplastic heat exchangers could create a reality where transparent scaled‐down heat exchangers are made as models of larger opaque heat exchangers to visualize how fluid flows and mixes inside a given heat exchanger and improve performance with design modifications. The potential for future applications motivated our approach to retain only transparent thermoplastics for our experiments in order enable these capabilities. We visualize flow regimes in our tube‐on‐tube heat exchangers in Figure 2c and in Movie S1, with a straight laminar stream of dyed fluid and turbulent cone of dyed fluid representing each regime. This visualization technique provides an opportunity for visual confirmation of changing flow regimes, and, notably, it displays similar results to the seminal work by Osborne Reynolds, with his figures reproduced in Figure 2e for comparison [38].

#### 2.2.5 Increasing Conduction Resistance Effect on Performance

**FIGURE 3:**
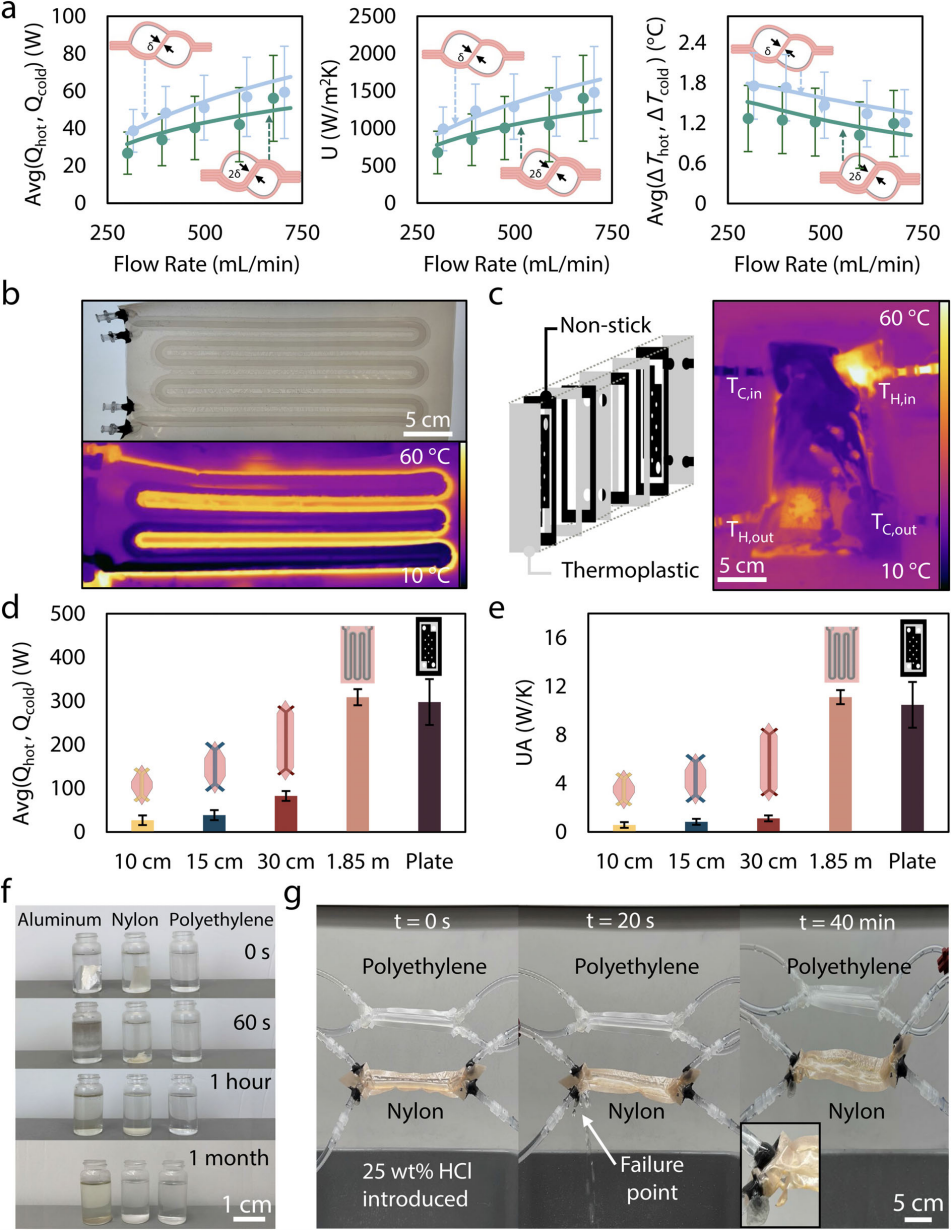
Sheet‐based thermal management variations. (a) Doubled conduction resistance demonstrates a limited impact on performance, demonstrating the dominance of convection resistance. (b) Scaled serpentine tube‐on‐tube design in the visible and IR spectrum under parallel flow. (c) Average rate of heat transfer of the scaled designs of the serpentine tube‐on‐tube and plate heat exchangers versus the fundamental tube‐on‐tube designs. (d) The sheet‐based technique applied to a prevalent industrial design, the plate heat exchanger (exploded view and visible and IR spectrum images). (e) Performance in terms of *UA* showing the increase in heat transfer capability with the scaled versions of the heat exchangers. (f) Chemical resistance testing using 25 wt.% hydrochloric acid (HCl) on three common material sheets of ∼50 µm thickness, with polyethylene showing no degradation over a period of 1 month. (g) Testing the resistance of thermoplastics to the 25 wt.% acidic solution of HCl under live operation, resulting in failure of the non‐acid resistant polymer nylon within 20 s of the introduction of the solution while polyethylene continued to perform indefinitely in the corrosive environment.

In the thermal resistance network in Figure 1d, the resistor is made up of three components: two convective terms and one conductive term. Figure 2 shows the effect of varying convection resistance, as the changing flow rate results in fluctuating convection terms. Similarly, we set out to investigate a variation of the conduction term by varying the thickness of the internal layer (δ). We expect that a 2x increase in δ will result in less than 2x reduction in performance, due to the dominating effects of the convection terms in the resistance network (Section S7). The dominance of convection resistance demonstrates the viability of the low thermal conductivity thin polymeric sheets for other heat transfer applications in future work, and also indicates that future work should focus on reducing the convection resistance rather than seeking higher thermal conductivity structural materials. We fabricated tube‐on‐tube heat exchangers with a 15 cm length, a 56% overlap fraction, and two stacked middle layers resulting in a thickness of 2δ. The results in Figure 3a indicate a 22% reduction of all performance characteristics (*Q*, Δ*T*, and *U*) on average, demonstrating a drop off in performance substantially smaller than 0.5x when doubling δ. Additionally, the analytical models detailed in Section 2.2.1 match the trends shown experimentally.

### 2.3 Variations of Sheet‐Based Thermal Management Designs

#### 2.3.1 Scaled Serpentine Tube‐on‐Tube Heat Exchanger

One of the most important aspects of this research is to outline the scalability of the work for future applications. We undergo this process by scaling our centimeter‐scale tube‐on‐tube heat exchangers to the meter scale (∼2 m). Increasing the length of the heat exchanger subsequently increases the area of heat transfer for the device, which is one of the main factors in the overall rate of heat transfer between the two streams. We then fabricate the scaled design through a serpentine pattern of 56% overlapping channels in order to demonstrate the scalability of performance in terms of *Q*, Δ*T*, and *U*. The serpentine design with 3 turns enables scaling to nearly 2 meters of length in a compact footprint of less than 350 cm^2^. The heat exchanger has sufficient length to visualize the Δ*T* across the device, shown in Figure 3e with more muted colors at the top of the IR image (corresponding to equilibration in temperature of the two steams) compared to the more vibrant colors on the bottom of the IR image demonstrating a temperature difference of ∼16°C. The IR images are solely for visualization, as all data is collected via thermocouples outlined in the Experimental Section. Visualizing the performance of this serpentine tube‐on‐tube device, we hold the flow rate constant at 300 mL/min and compare the rate of heat transfer to other devices from this work at the same 300 mL/min flow rate for a fair comparison. The increase in performance of the serpentine heat exchanger is apparent in Figure 3d with *Q* increasing by 8x and in Figure 3e with *UA* increasing by 14x on average across the 10, 15, and 30 cm designs.

#### 2.3.2 Plate Heat Exchanger

The preceding analyses in this work have been centered on the tube‐on‐tube design, taking advantage of the compliance of our sheet‐based architecture to allow for such a design. The sheet‐based fabrication method leveraged in this work, however, is not limited to the tube‐on‐tube design and can be applied to other heat exchanger designs, including the quintessential plate heat exchanger. Prevalent in numerous industries, the plate heat exchanger is a scalable stack of plates that have a large surface contact area between two streams, with the streams typically flowing to the outlet diagonally from the inlet. For a sheet‐based device, additional non‐stick layers are necessary for the accordion‐like expansion of the plate and frame heat exchanger; the rest of the fabrication process remains the same as in Section 2.1, and Figure 3d details an exploded view of the plate heat exchanger. We expect the performance of the plate heat exchanger to maintain a high *UA* value compared to the centimeter‐scale heat exchangers, with a substantial increase in *Q*. Figure 3c–e compare the plate heat exchanger results, and *Q* and *UA* increase by 7x and 13x on average across the 10, 15, and 30 cm designs.

#### 2.3.3 Acid‐Resistant Tube‐on‐Tube Heat Exchanger

Polymer heat exchangers are known for their chemical compatibility, affordability, and corrosion and fouling resistance, but the selection of a specific polymer depends on an application's needs [13, 14, 15, 17, 40]. This work focuses on using thermoplastics and sheet‐lamination techniques to form heat exchangers; additionally, we seek to demonstrate the chemical compatibility of thermoplastics that can withstand stress‐test scenarios relevant to the application of these devices. Polyethylene is a polymer known for electronics usage and chemical resistance to strong acids, leading to polyethylene being a primary candidate for our material selection for stress testing [8]. We chose hydrochloric acid (HCl) 25 wt.% concentration to highlight the chemical resistance potential of sheet‐based thermoplastics like polyethylene, and we compared the polyethylene device to a nylon device (representing the other polymer prominently used in this work). Additionally, we compare these two thin (<100 µm) polymer sheets to a similarly thin metal aluminum foil sheet to test a metallic material common in heat exchangers. Figure 3f details an exposure experiment where three equally sized samples are placed into a solution of HCl 25 wt.% concentration and exposed to the solution for more than one month. The nylon and aluminum sheets begin to dissolve within one minute of being placed into the vials and fully dissolve within one hour. During the same time period, the polyethylene sheet remains fully intact and resistant to the acidic solution, and in fact, it remains in the solution for one month with no sign of material degradation. To test the application of this material advantage, we fabricated a polyethylene heat exchanger with a design identical to the previously used nylon heat exchanger (in this case, with 15 cm length and 56% overlap) and set up an experiment to demonstrate its performance compared to a non‐chemically compliant nylon heat exchanger, as shown in Movie S2 and Figure 3g. A variety of materials are not compatible with acids like HCl, so our results from a non‐compatible device like the nylon heat exchanger can be extrapolated to a number of other non‐compatible materials of similar wall thicknesses that could be used in thermal engineering. The epoxies used in sealing the ends of the polymer heat exchangers are chemically compatible in short periods of time, so failure will not be related to the epoxies themselves. Beginning with DI water as the working fluid, both heat exchangers operate on separate peristaltic pumps with polyethylene tubing to ensure that there is no degradation or failure in the testing apparatus. In Figure 3g, 37 wt.% HCl is then introduced to the reservoir connected to the heat exchangers which had initially been composed of solely pure DI water to create a known solution concentration of 25 wt.% HCl. Within 20 s of exposure, failure occurs in the non‐compatible nylon heat exchanger near the point of entry to the system resulting in a large amount of leakage. After 40 min from initial exposure, the nylon heat exchanger shows severe material degradation and numerous holes that violate its initially hermetic operation. The polyethylene heat exchanger simultaneously remains fully hermetic throughout the test, and after 40 min from the initial exposure demonstrates no visible degradation.

### 2.4 Single‐Channel Thermal Management Device

Our sheet‐based thermal management devices can be applied beyond fluid‐fluid heat exchange to single channel pumped fluid loops. This type of active thermal management system can be applied to a variety of applications, including cooling of electronics, bulk fluid heating, and wearable cooling. We investigate two of these applications: ambient water bath heating and a wearable cooling solution for recovery from labor‐intensive action or exercise. Both applications include IR visualizations for clarity and report all temperature data via thermocouples or heat flux sensor as outlined in the materials and methods section.

#### 2.4.1 Water Bath Bulk Fluid Heating

**FIGURE 4:**
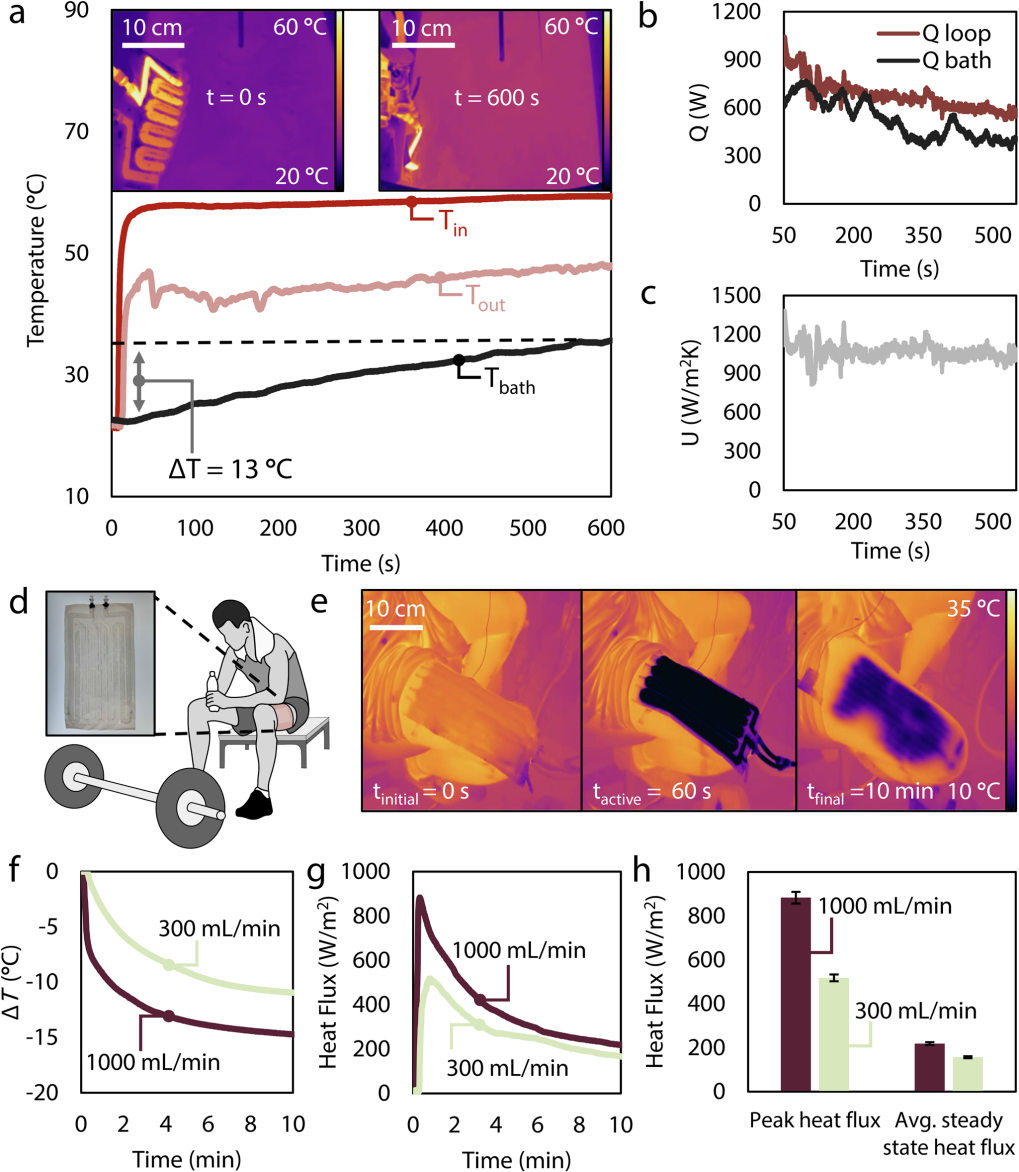
Single‐channel immersion chiller or heater. (a) Water bath warming demonstration of the single‐channel device, demonstrating an increase of 13°C in 10 min. (b) The rates of heat transfer of the fluid in the loop and of the bath, decreasing over time as the Δ*T* between the bath and the fluid loop decreases. (c) The overall heat transfer coefficient *U* remains stable throughout the process despite the decrease in Δ*T* between the bath and the fluid loop. (d) A wearable patch for personal cooling shown via a photo and a schematic of its intended use. (e) IR image results of application, activation, and aftereffect of the wearable cooling patch, with a decrease of up to 15°C from the initial body surface temperature. (f) The decrease in temperature from the initial body temperature to the final body temperature at two different flow rates resulting in Δ*T* of 11°C and 15°C, respectively, shown over time. (g) Heat flux versus time showing an early peak and a slow decrease with time as body surface temperature decreases. (h) The peak and average steady state heat flux of both flow streams.

The bulk heating and cooling of fluids as an application of thermal management goes back more than two millennia. For example, Roman hypocausts (i.e., underground furnaces) would heat water for Roman public baths and warm homes, a precursor technology to modern central heating systems [41]. We simulate a scenario for our devices to either cool or heat bulk fluids, where today the applications can range from data center cooling to wort chilling in malt beverage production. Beginning with a water bath of 5 L of DI water at room temperature (22°C), we set up a mixer spinning at 360 RPM to ensure a thorough distribution of the fluid once it undergoes heating or cooling, but not so fast as to impact the temperature of the bulk fluid. Additionally, we measure the temperature through IR visualization and a thermocouple inside the bulk fluid to capture the change in temperature with time. We set up our single‐channel 5‐turn serpentine device with thermocouples at the inlet and the outlet of the device, along with a flow meter to verify the set 700 mL/min flow rate. We exposed the bulk fluid bath at 22°C to a 60°C inlet temperature fluidic channel temperature for 10 min and observed a 13°C increase in temperature of the bath (Figure 4a). As the fluid bath temperature steadily increases to 35°C, the outlet temperature of the single‐channel heater increases due to the decrease in Δ*T* between the inlet temperature and the fluid bath. Figure 4b also shows a decrease in *Q* because both *Q* and Δ*T* decrease with time when the fluid bath temperature increases. We expect the overall heat transfer coefficient to remain more stable throughout the testing period, as the fluidic parameters (heat capacity and flow rate) are larger contributors to this term than the Δ*T* between the bath and the fluid stream (Figure 4c).

#### 2.4.2 Wearable Cooling Patch

One of the primary target applications of a single‐stream loop as a system is for wearable cooling for athletic or labor‐intensive work recovery; this type of recovery solution has been shown to be effective in the literature [42, 43, 44, 45, 46]. We hypothesized that our device would work well for this cooling application. We used the single‐channel serpentine design and attached the device to a person for a cooling session as depicted in Figure 4d. Our approach using the sheet‐lamination technique could lend itself to greater integration with wearable textiles and thus avoid the cumbersome nature of more‐rigid tubing. Additionally, the compliance of the inflated channel allows for better contact with the user for more effective heat exchange. The fluid in our chiller was set to 10°C and we attached our device with medical tape to a subject's leg for two trials with two distinct flow rates: 300 mL/min and 1000 mL/min. After 10 min of exposure, the individual's surface temperature was reduced by 11°C and 15°C, respectively (Figure 4f), all within the pain tolerance of the individual, as shown in a series of photos in Figure 4e. The peak heat flux of 883 W/m^2^ occurred during the 1000 mL/min flow, greater than the 300 mL/min peak, but the average heat fluxes for these two cases get closer to each other after 10 min (Figure 4g,h). The changes in temperature from the initial body temperature demonstrate that the wearable cooling and heating application of our single‐channel device is well‐founded and shows promise for personal thermal management in addition to more traditional device thermal management.

## 3 Conclusion

Thermal management solutions of the future are necessary to help solve the world's energy needs, especially with the advent of the era of high‐power electronics, large data storage facilities, and computing centers for artificial intelligence. We present a thermal management approach focused on advancing polymer heat exchanger technology that can be scaled and ultimately utilized in these types of critical energy applications. By introducing a tube‐on‐tube sheet‐based architecture, we demonstrate the feasibility of a compliant, optically transparent heat exchanger whose performance can be predicted via analytical modeling to enable application‐specific heat exchanger design and optimization. Our polymer heat exchangers serve as an effective thermal management system on their own, but may also find use as scaled‐down models of larger heat exchangers for operational understanding and iteration. This work centers on polymer materials, which offer distinct advantages compared to metal counterparts, but these materials still can benefit from a higher thermal conductivity applied similarly with sheet‐based fabrication; therefore, future work could involve hybrid doped materials of dispersed particles of metals or carbon‐based materials inside a bulk polymer matrix to increase the thermal conductivity of the material, further driving down the conduction resistance while maintaining many of the advantages of polymeric materials. Additional future work can use the sheet‐based architecture for multiphase flow, with both liquid and vapor states present, to expand the applications of this sheet‐based platform to two‐phase thermal systems that require deeper characterization [47]. Ultimately, this work provides a basis for innovation and deeper characterization in compliant sheet‐based polymeric heat exchangers, which can be made through a variety of techniques for the next generation of thermal management systems.

## 4 Experimental Section

### 4.1 Flow Visualization

We visualize fluid flow by inserting a needle parallel to the flow direction through the wall of the thermoplastic material comprising the heat exchanger. The compliance of the heat exchanger when the system is active allows for the insertion of a sharp needle and syringe where necessary in the system, enabling flow visualization at a multitude of points throughout the system. We undergo flow visualization in the laminar regime for a 10°C flow at 300 mL/min, and a 60°C turbulent flow at 1000 mL/min. The base fluid remains DI water, and the dyed fluid is DI water dyed with black water‐based food‐grade dye.

### 4.2 Experimental Setup and Data Collection

All heat exchangers are tested in the same experimental setup, shown in Figure S2. A flowmeter (OMEGA FTB2004‐C) and needle valve (McMaster 46425K12) are used alongside the data collection system for each fluid stream to measure the flow rate and control it throughout the experiment. Additionally, a custom MATLAB code live plots the flow rate to allow for flow rate adjustment in between trials with the needle valves, allowing for a high level of tunability. Calibrated OMEGA T‐type thermocouple probes are placed directly inside the inlets and outlets of the polymer heat exchanger or the single‐channel chiller or heater via a Swagelok probe compression fitting and sampled 10 times per second. This data is also plotted live and collected through the same code for the flow rate. A heat flux sensor (FluxTeq, PHFS‐01) was used for the single‐channel wearable cooling application. A calibrated pressure transducer with a range reaching vacuum pressure (UPC 760385568020)—used for accurate readings with a force and suction pump like the circulating bath (Thermo Scientific NESLAB RTE 7)—is able to read the pressure drop across the heat exchanger, further detailed in Section S2. We used two peristaltic pumps (Kamoer KPHM400‐SW3B25) for the corrosive fluid demonstration, adding hydrochloric acid 37 wt.% to DI water for an operational concentration of 25 wt.% with a flow rate of 400 mL/min.

## Funding

DOE DE‐EE0011230, DoD SMART Scholarship, NSF GRFP Fellowship

## Conflicts of Interest

B.J. and D.J.P. declare an equity interest in Actile Technologies, Inc., a company commercializing wearable devices including for personal thermal management.

## Supporting information




**Supporting File 1**: advs74114‐sup‐0001‐SuppMat.pdf.

**Supporting File 2**: advs74114‐sup‐0002‐MovieS1.mp4.

**Supporting File 3**: advs74114‐sup‐0002‐MovieS1.mp4.

## Data Availability

The data that support the findings of this study are available in the supplementary material of this article.

## References

[1] H. Ritchie , P. Rosado , and M. Roser , “Energy Production and Consumption, Our World in Data,” 2022.

[2] IEA, Renewables 2023 2024.

[3] C. Geffroy , D. Lilley , P. S. Parez , and R. Prasher , “Techno‐Economic Analysis of Waste‐Heat Conversion,” Joule 5 (2021): 3080–3096, 10.1016/j.joule.2021.10.014.

[4] K. Thulukkanam , Heat Exchangers: Classification, Selection, and Thermal Design. (CRC Press, 2024).

[5] J. G. Cevallos , A. E. Bergles , A. Bar‐Cohen , P. Rodgers , and S. K. Gupta , “Polymer Heat Exchangers—History, Opportunities, and Challenges,” Heat Transfer Engineering 33 (2012): 1075–1093, 10.1080/01457632.2012.663654.

[6] H. T. El‐Dessouky and H. M. Ettouney , “Plastic/Compact Heat Exchangers for Single‐Effect Desalination Systems,” Desalination 122 (1999): 271–289, 10.1016/S0011-9164(99)00048-X.

[7] T. Liu and M. S. Mauter , “Heat Transfer Innovations and Their Application in Thermal Desalination Processes,” Joule 6 (2022): 1199–1229, 10.1016/j.joule.2022.05.004.

[8] X. Chen , Y. Su , D. Reay , and S. Riffat , “Recent Research Developments in Polymer Heat Exchangers—A Review,” Renewable and Sustainable Energy Reviews 60 (2016): 1367–1386, 10.1016/j.rser.2016.03.024.

[9] E. D. Stevens , Encyclopedia of Fish Physiology. (Elsevier, 2011): 1119–1131.

[10] L. Li , M. Liang , B. Yu , and S. Yang , “Analysis of Thermal Conductivity in Living Biological Tissue with Vascular Network and Convection,” International Journal of Thermal Sciences 86 (2014): 219–226, 10.1016/j.ijthermalsci.2014.07.006.

[11] R. Githens , R. Minor , and V. Tomsic , “Flexible‐Tube Heat Exchangers,” Chemical Engineering Progress 61 (1965): 55–62.

[12] D. A. Reay , “The Use of Polymers in Heat Exchangers,” Heat Recovery Systems and CHP 9: 209–216, 10.1016/0890-4332(89)90004-5.

[13] S. N. Kazi , G. G. Duffy , and X. D. Chen , “Mineral Scale Formation and Mitigation on Metals and a Polymeric Heat Exchanger Surface,” Applied Thermal Engineering 30 (2010): 2236, 10.1016/j.applthermaleng.2010.06.005.

[14] W. A. Laftah and W. A. W. Abdul Rahman , “Polymers for Anti‐Fouling Applications: A Review,” Environmental Science: Advances 4 (2025): 824–841.

[15] J.‐H. Imholze and H. Glade , “Crystallization Fouling on Polymer Composite Heat Exchanger Tubes and the Effects of Surface Treatments,” Heat and Mass Transfer 61: 78, 10.1007/s00231-025-03596-y.

[16] Z. Liu , R. M. Rasheed , A. Rajappan , et al., “Scalable Hot‐Water‐Repellent Superhydrophobicity via Thermal Insulation,” ACS Applied Materials & Interfaces 18 (2026): 4401–4412. 41511007 10.1021/acsami.5c17943 PMC12829580

[17] D. C. Deisenroth , R. Moradi , A. H. Shooshtari , F. Singer , A. Bar‐Cohen , and M. Ohadi , “Review of Heat Exchangers Enabled by Polymer and Polymer Composite Additive Manufacturing,” Heat Transfer Engineering 39 (2018): 1648–1664, 10.1080/01457632.2017.1384280.

[18] S. A. Niknam , M. Mortazavi , and D. Li , “Additively Manufactured Heat Exchangers: A Review on Opportunities and Challenges,” The International Journal of Advanced Manufacturing Technology 112 (2021): 601–618, 10.1007/s00170-020-06372-w.

[19] I. Kaur and P. Singh , “State‐of‐the‐art in Heat Exchanger Additive Manufacturing,” International Journal of Heat and Mass Transfer 178 (2021): 121600, 10.1016/j.ijheatmasstransfer.2021.121600.

[20] A. Rajappan , B. Jumet , R. A. Shveda , et al., “Logic‐Enabled Textiles,” Proceedings of the National Academy of Sciences 119 (2022): 2202118119, 10.1073/pnas.2202118119. PMC9436326 35994641

[21] F. Careri , R. H. U. Khan , C. Todd , and M. M. Attallah , “Additive Manufacturing of Heat Exchangers in Aerospace Applications: A Review,” Applied Thermal Engineering 235 (2023): 121387, 10.1016/j.applthermaleng.2023.121387.

[22] I. Gibson , D. W. Rosen , and B. Stucker , Additive Manufacturing Technologies (Springer US, 2010): 223–252, 10.1007/978-1-4419-1120-9.

[23] B. Jumet , Z. A. Zook , A. Yousaf , et al., “Fluidically Programmed Wearable Haptic Textiles,” Device 1 (2023): 100059, 10.1016/j.device.2023.100059.

[24] M. D. Bell , K. Ye , T. F. Yap , et al., “Rapid In Situ Thermal Decontamination of Wearable Composite Textile Materials,” ACS Applied Materials & Interfaces 15 (2023): 44521, 10.1021/acsami.3c09063. 37695080 PMC10521748

[25] N. Fino , B. Jumet , Z. A. Zook , D. J. Preston , and M. K. O'Malley , “Mechanofluidic Instability‐Driven Wearable Textile Vibrotactor,” IEEE Transactions on Haptics 16 (2023): 530, 10.1109/TOH.2023.3271128. 37104109

[26] V. T. Vo , A. Rajappan , B. Jumet , M. D. Bell , S. Urbina , and D. J. Preston , “Sheet‐Based Fluidic Diodes for Embedded Fluidic Circuitry in Soft Devices,” Advanced Intelligent Systems 6 (2024): 2300785, 10.1002/aisy.202300785.

[27] T. F. Yap , J. Klinkao , S. Urbina , et al., “Understanding Silicone Elastomer Curing and Adhesion for Stronger Soft Devices,” Science Advances 11 (2025): adv2681, 10.1126/sciadv.adv2681. PMC12266107 40668914

[28] A. Broshkevitch , S. Urbina , B. Jumet , J. A. Garavito‐Leon , A. Rajappan , and D. J. Preston , “Programmable Failure in Heat‐Sealable Sheet‐Based Fluidic Devices,” Cell Reports Physical Science 6 (2025): 102437, 10.1016/j.xcrp.2025.102437.

[29] D. Zheng , J. Wang , Z. Chen , J. Baleta , and B. Sundén , “Performance Analysis of a Plate Heat Exchanger Using Various Nanofluids,” International Journal of Heat and Mass Transfer 158 (2020): 119993, 10.1016/j.ijheatmasstransfer.2020.119993.

[30] S. H. Noie , “Investigation of Thermal Performance of an Air‐to‐Air Thermosyphon Heat Exchanger Using ε‐NTU Method,” Applied Thermal Engineering 26 (2006): 559–567, 10.1016/j.applthermaleng.2005.07.012.

[31] T. L. Bergman , A. S. Lavine , F. P. Incropera , and D. P. DeWitt , Introduction to Heat Transfer, (Wiley, 2011).

[32] Y. S. Muzychka and M. M. Yovanovich , “Laminar Forced Convection Heat Transfer in the Combined Entry Region of Non‐Circular Ducts,” Journal of Heat Transfer 126 (2004): 54–61, 10.1115/1.1643752.

[33] Y. Muzychka and M. Yovanovich , “Modeling Friction Factors in Non‐circular Ducts for Developing Laminar Flow,” 2nd AIAA, Theoretical Fluid Mechanics Meeting , (Albuquerque, NM, USA 1998): 2492.

[34] Y. Muzychka and M. Yovanovich , “Modeling Nusselt Numbers for Thermally Developing Laminar Flow in Non‐Circular Ducts,” 7th AIAA/ASME Joint Thermophysics and Heat Transfer Conference 1998: 2586.

[35] V. Gnielinski , “New Equations for Heat and Mass Transfer in Turbulent Pipe and Channel Flow,” International Chemical Engineering 16 (1976): 359–397.

[36] A. P. C. Sarmiento , V. H. T. Soares , F. H. Milanez , and M. B. H. Mantelli , “Heat Transfer Correlation for Circular and Non‐Circular Ducts in the Transition Regime,” International Journal of Heat and Mass Transfer 149 (2020): 119165, 10.1016/j.ijheatmasstransfer.2019.119165.

[37] A. P. C. Sarmiento , F. H. Milanez , and M. B. H. Mantelli , “Theoretical Models for Compact Printed Circuit Heat Exchangers with Straight Semicircular Channels,” Applied Thermal Engineering 184: 115435, 10.1016/j.applthermaleng.2020.115435.

[38] O. Reynolds , “XXIX. An Experimental Investigation of the Circumstances Which Determine Whether the Motion of Water Shall be Direct or Sinuous, and of the Law of Resistance in Parallel Channels,” Philosophical Transactions of the Royal Society of London 174 (1883): 935–982, 10.1098/rstl.1883.0029.

[39] R. V. Crystal , T. Crittenden , A. Glezer , and S. Garimella , “Enhancement of Air‐side Heat Transfer in Crossflow Heat Exchangers Using Auto‐fluttering Reeds,” Applied Thermal Engineering 258 (2025): 124617, 10.1016/j.applthermaleng.2024.124617.

[40] D. M. Zarkadas and K. K. Sirkar , “Polymeric Hollow Fiber Heat Exchangers: An Alternative for Lower Temperature Applications,” Industrial & Engineering Chemistry Research 43 (2004): 8093–8106, 10.1021/ie040143k.

[41] L. C. McParland , Z. Hazell , G. Campbell , M. E. Collinson , and A. C. Scott , “How the Romans Got Themselves into Hot Water: Temperatures and Fuel Types Used in Firing a Hypocaust,” Environmental Archaeology 14 (2009): 176–183, 10.1179/146141009X12481709928445.

[42] J. Ingram , B. Dawson , C. Goodman , K. Wallman , and J. Beilby , “Effect of Water Immersion Methods on Post‐Exercise Recovery from Simulated Team Sport Exercise,” Journal of Science and Medicine in Sport 12 (2009): 417–421, 10.1016/j.jsams.2007.12.011. 18547863

[43] T. R. Higgins , D. A. Greene , and M. K. Baker , “Effects of Cold Water Immersion and Contrast Water Therapy for Recovery from Team Sport: a Systematic Review and Meta‐analysis,” Journal of Strength and Conditioning Research 31 (2017): 1443–1460, 10.1519/JSC.0000000000001559. 27398915

[44] P. Kotagama , A. Phadnis , K. C. Manning , and K. Rykaczewski , “Rational Design of Soft, Thermally Conductive Composite Liquid‐Cooled Tubes for Enhanced Personal, Robotics, and Wearable Electronics Cooling,” Advanced Materials Technologies 4 (2019): 1800690, 10.1002/admt.201800690.

[45] P. Kotagama , K. C. Manning , and K. Rykaczewski , “Fundamentals of Soft Thermofluidic System Design,” Soft Matter 16 (2020): 6924–6932, 10.1039/D0SM00504E. 32686814

[46] J. Yang , Y. Xiong , J. Piao , et al., “Wearable Fluidic Fabric with Excellent Heat Transfer Performance for Sports Recovery,” Advanced Science 12 (2025): 2411691. 39764745 10.1002/advs.202411691 PMC11848550

[47] R. J. Fontenot , D. J. Lockwood , J. M. Allison , and D. J. Preston , “A Review and Outlook on Osmotically Driven Heat Pipes for Passive Thermal Transport,” Applied Thermal Engineering 248 (2024): 123097.

