# Supplementary material for: Compliant Polymeric Sheet‐Based Heat Exchangers
Source: Adv Sci (Weinh). 2026 Feb 21;13(19):e20009. doi: 10.1002/advs.202520009 (PMC13045345; doi:10.1002/advs.202520009)
Supplement: Supplementary file 1 — Supporting File 1: advs74114‐sup‐0001‐SuppMat.pdf. [file ADVS-13-e20009-s002.pdf]

Supporting Information

# Compliant Polymeric Sheet-based Heat Exchangers

*Richard J. Fontenot, Loic Duggal, Sofia Urbina, Barclay Jumet, Anoop Rajappan, and Daniel J. Preston**

Richard J. Fontenot, Loic Duggal, Sofia Urbina, Barclay Jumet, Prof. Daniel J. Preston
Department of Mechanical Engineering, Rice University, Houston, USA

Prof. Anoop Rajappan
Department of Physics and Engineering Physics, Tulane University, New Orleans, USA

Prof. Daniel J. Preston
Rice Advanced Materials Institute, Rice University, Houston, USA
Rice Space Institute, Rice University, Houston, USA
Carbon Hub, Rice University, Houston, USA
*E-mail: djp@rice.edu

**Section S1: Fabrication and Experimental Setup**

***Fabrication method***

In this work, we use a sheet-based lamination fabrication method, where thin sheets of materials are aligned and bonded together through the application of heat and pressure. First, we create the designs in a computer-aided design (CAD) software, specifically designed to include prescribed overlap fractions and alignment tabs if necessary. Then we utilize a vinyl cutter, the Cricut Maker 3, to selectively cut out the polymer material with a sharp blade to preserve the integrity of the material, because alternative techniques like laser cutting have the potential to degrade the material. Subsequently, the layers are aligned with a non-stick layer (CS Hyde UHMW Polyethylene Tape) outlining the prescribed channel or fluidic area of the device. This non-stick layer is oriented on the side of the tube that does not play a role in heat transfer, so as to not add conduction resistance across the thin wall between the two streams. Alternatively, the masking layer can be removed easily if a water soluble non-stick material that can be dissolved is selected.[1] We carefully align each layer, especially for the tube-on-tube design where an overlap fraction is determined based on the channel flat length of 1 cm, and therefore the overlap fraction is simply the decimal equivalent in units of cm. For example, a 56% overlap is a 0.56 cm overlap between the two non-stick layers. We set this alignment in a CAD software, and the non-stick tape layer is used with the adhesive backing maintain an accurate placement onto the thermoplastic as the layers are aligned for the next step, heat pressing. The heat exchanger undergoes a global heat pressing process, where the entire device is placed under high heat and pressure and the thermoplastics reflow and bond each other. We bond the nylon material (Fibre Glast Stretchlon 800) at 207 °C and 3.5 bar for 30 seconds, and we bond the polyethylene (Fibre Glast Polyethylene Bagging Film)—used for the acid corrosion experiment described in Section 2.3.4 in the main text and shown in **Figure 3** and **Movie S2**—at 125 °C and 3.5 bar for 30 seconds. We then remove each bonded device from the heat press and let it cool under a heavy metal plate in a cold pressing step because the device after cold pressing exhibits less material warping on the edges than if allowed to fully cool without an applied pressure. After cooling, we epoxy the device with Luer-lock fittings on the inlets and outlets to allow for connection to the tubing and sensors for operation. After the epoxy cures, the finished heat exchanger is ready for attachment to the testing apparatus for operation.

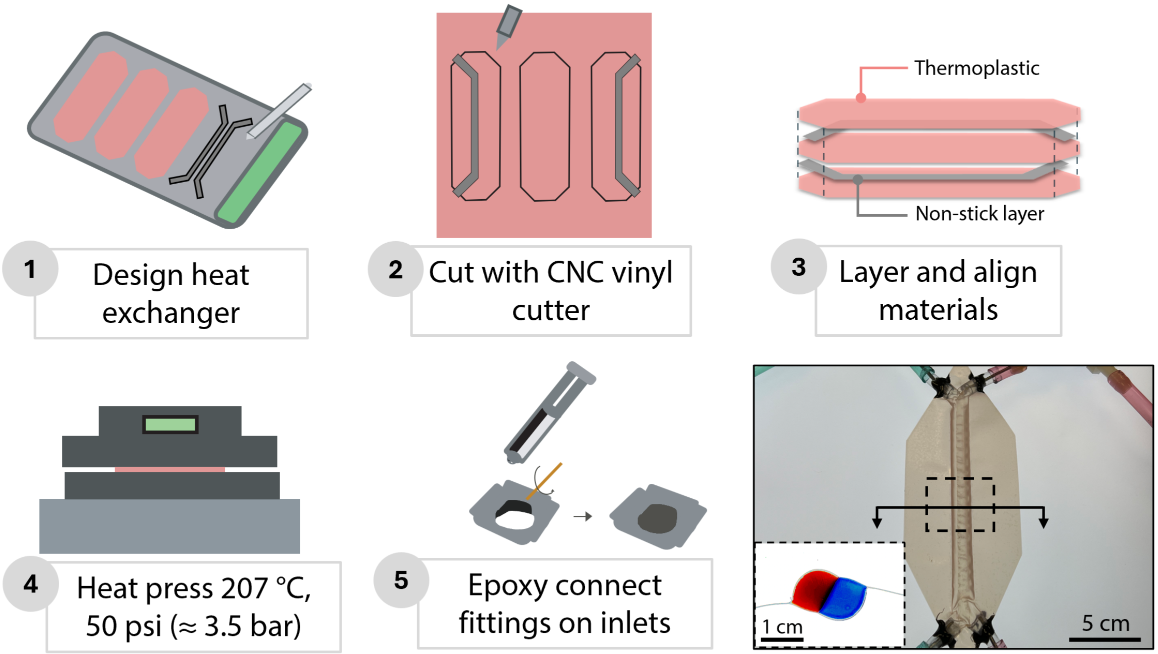


**Figure S1.** Fabrication steps to create sheet-based heat exchangers. The inset image in the bottom-right panel was obtained by injecting pressurized uncured liquid elastomer into each flow channel of the heat exchanger, with red and blue elastomer dye used to visually set apart the hot and cold streams, and then allowing the elastomer to cure before physically cross-sectioning and imaging the slice of the heat exchanger shown here with backlighting for visualization.

***Experimental setup***

The heat exchanger testing process begins with the tabletop bench setup which has four cross-fittings, two prescribed to the flow-path, and the other two prescribed to the pressure drop sensors and thermocouples probe as shown in **Figure S2b**. We use two recirculating baths (NESLAB RTE-7) to control the given inlet temperatures throughout the entire testing process to ensure that the losses as the test runs remain at a minimum, and we obtained our pump curve (**Figure S3**) from the pump's documentation. We use two setups throughout this work, one for parallel flow and one for counterflow additionally detailed in **Figure S2b**. The temperature probes are T-type thermocouples that are accurate within 0.1 °C or 1% and are read through a National Instruments Data Acquisition (NI DAQ) device recording 10 samples per second. The pressure sensors record the pressure at the inlet and outlet, and the change in pressure is recorded by taking the difference between them. **Figure S5** shows the results of the pressure drop for the tube-on-tube design, where

the increasing flow rate and length result in a higher pressure drop than expected. A flowmeter records the volume passing through the device, subsequently converting the volume allowed through with the time elapsed over 10 points within 1 second periods and with this data we can determine the volumetric flow rate. Additionally, needle valves control the flow rate for fine-tuning of the designed flow rate set point. The pressure sensor and the flowrate are recorded through a DAQ device separate from the thermocouple sensor which is on a separate DAQ. Lastly, we live-plot all data through a MATLAB script for operational observation of temperature, flowrate, and pressure difference.

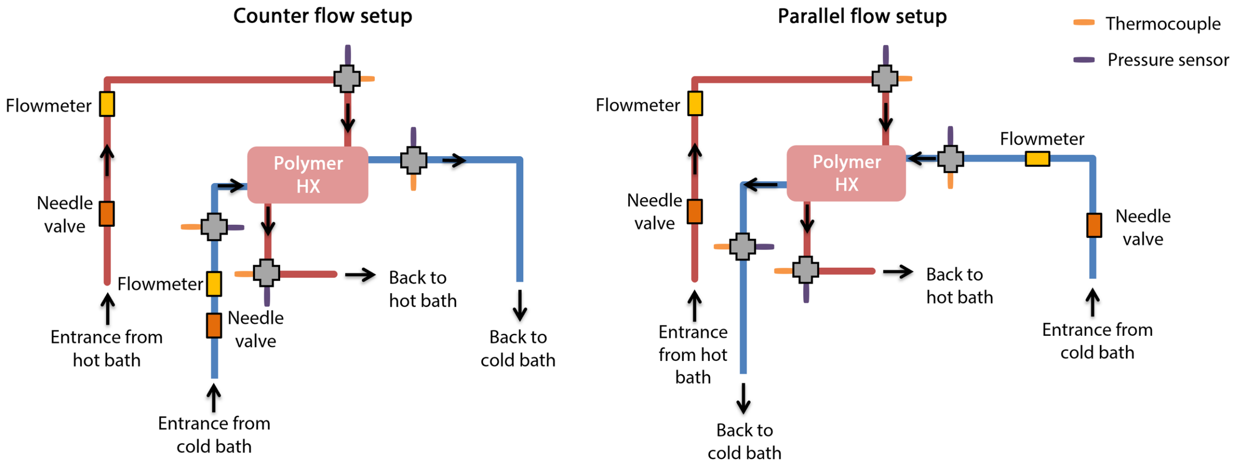


**Figure S2.** Top-view graphical experimental setup with the orientation of the flowmeters with the needle valves and the close proximity of the thermocouples to the heat exchanger. In reality, the thermocouple probes reach slightly inside the entrances and exits of the heat exchanger to ensure minimal temperature reading error.

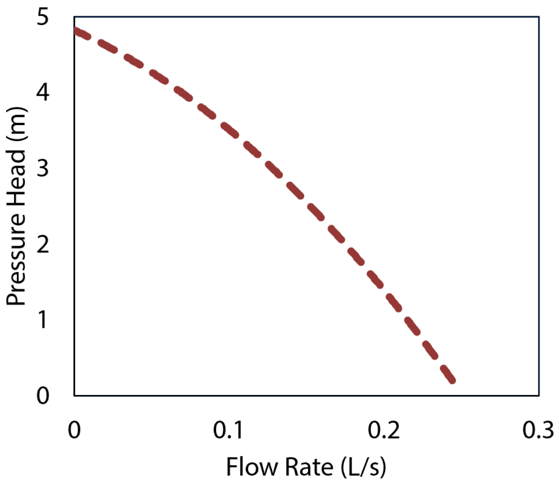


**Figure S3.** Pump curve for the recirculating pump baths used in this work, obtained and digitized from the NESLAB RTE-7 documentation.

## Section S2: Pressure Drop and Temperature Loss

***Pressure drop in tube-on-tube heat exchangers***

One of the major considerations for development of heat exchangers is the trade-off of improved heat exchange capacity of a novel design and the increase in pressure drop that can accompany it. Determining the pressure drop for a system is essential for characterization; for our tube-on-tube heat exchangers, pressure transducers (UPC 760385568020) are attached to the four-way fittings and measure the pressure at the inlet and the outlet of each stream. The pressure transducers were calibrated independently. The pressure readings are taken and recorded through a DAQ device and MATLAB code and converted to a change in pressure for the three compact heat exchanger lengths of 10 cm, 15 cm, and 30 cm (Figure S4). The pressure drop at different flow rates was expected to be noticeably different, and we see this increase in pressure drop as the flow rate increases along with an increase as a function the length of the device as well. The pressure drops in our system are comparable to those in other works, such as $\Delta P$ ~ 0.1 bar at Re = 1000 for the GAF device from H. Moon et al., and $\Delta P$ ~ 0.2 bar for Re ~ 11500 from L. Hein and M. Mortean, indicating that pressure drop is not a limiting factor in our approach.[2,3]

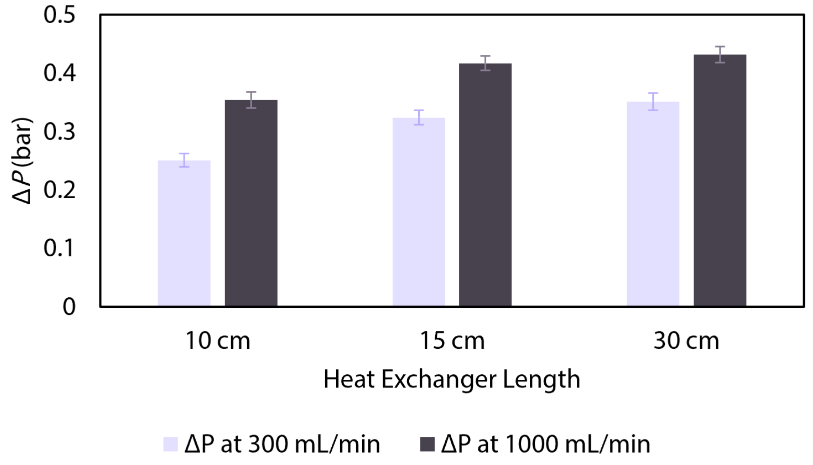


**Figure S4.** Pressure drop comparison for the compact tube-on-tube heat exchangers at 10 cm, 15 cm, and 30 cm lengths.

***Losses to the environment***

We analyze heat transfer with the environment to ensure that the $\Delta T$ measured from the inlet to outlet of each stream in our experiments is primarily due to the exchange of heat between the fluids in the two streams and not heat exchange with the ambient. We conducted tests on the 30 cm tube-on-tube design at both at 1000 mL/min and 300 mL/min, with a modification to our typical procedure such that we either provided an inlet temperature of 60 °C to both channels at the same time (and therefore no heat exchange occurred from one channel to the other, isolating the effect of heat exchange with the ambient; labeled "hot flow" in Figure S5), or provided an inlet temperature of 10 °C to both channels at the same time (labeled "cold flow" in Figure S5), where 60 °C and 10°C represent the maximum and minimum temperatures utilized in this study. The results show temperature changes of < 0.15 °C from the inlet to the outlet for these streams under exposure to an ambient temperature between 21 °C to 22 °C. These temperature changes are acceptable given the $\Delta T$ values from inlet to outlet of each stream from our experimental heat exchanger tests are all over 1 °C, and therefore the heat losses (or gains) due to thermal exchange with the ambient are neglected in this work.

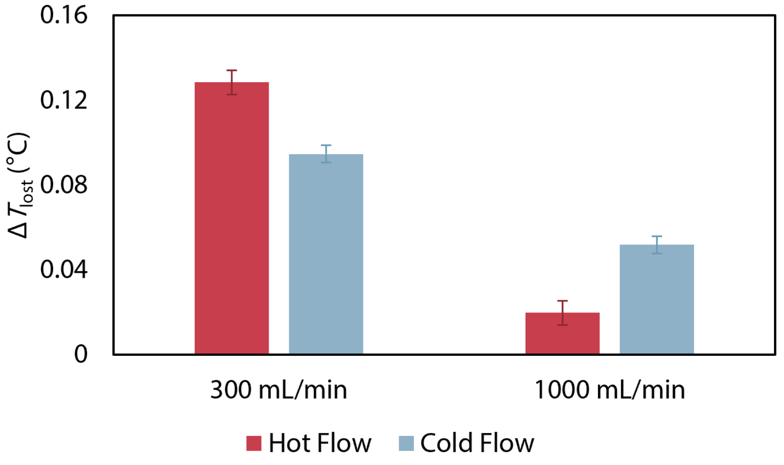


**Figure S5.** Change in temperature from inlet to outlet of hot flow (60 °C ) and cold flow (10 °C) in both channels simultaneously (i.e., no heat exchange between channels). The 300 mL/min and 1000 mL/min flow rates are the slowest and fastest flow rates tested in this work. The hot flow is at 60 °C and the cold flow is at 10 °C, corresponding to the maximum and minimum temperatures of the inlet streams in this work, respectively. The error bars represent the standard deviation of 1,200 total data points from two sets of thermocouple data of inlet and outlet temperatures.

## Section S3: Literature Comparison

### ***Literature comparison to metal and polymer heat exchangers***

We compare our results for the compact tube-on-tube and the scaled serpentine and plate heat exchangers to relevant studies from the heat exchanger literature in **Table S1**. We select three polymer heat exchangers and three metal heat exchangers that provide the relevant information needed to compare our devices in terms of performance and material cost estimates. The limited number of papers selected is due to the literature not providing either the relevant cost estimates of fabrication or materials or the performance in terms of overall heat transfer coefficients $U$ and $UA$, both of which were needed in order for us to include the reference in **Figure 1h** in the main text. The works shown in the comparison table have their respective assumptions and estimation process outlined below **Table S1** as a list of notes. In general, our work provides an effective $UA$ performance for a given material cost, not accounting for the even greater savings in fabrication equipment cost. The sheet-based heat exchangers allow for exceptional performance at a low cost. Additionally, we expand our comparison to the literature in **Figure 1h** in **Table S2**, that includes more polymer heat exchanger papers with their U values reported.

**Table S1.** Comparison of this work to the literature. Cost data and values are estimated based on provided information in the main text and SI of the respective works and material cost data available at the time of publication. Notes indicated by superscripts appear following the table, on the next page.

| Author | HX design | Cost estimate ($USD) | U ($W/m^2K$) | UA (W/K) | UA/cost |
|---|---|---|---|---|---|
| This work[A] | Tube-on-tube 10 cm (300 mL/min) | 0.36 | 1023.1 | 0.57 | 1.57 |
| | Tube-on-tube 15 cm (300 mL/min) | 0.43 | 992.4 | 0.83 | 1.95 |
| | Tube-on-tube 15 cm (500 mL/min) | 0.43 | 1289.5 | 1.08 | 2.53 |
| | Tube-on-tube 15 cm (700 mL/min) | 0.43 | 1479.4 | 1.24 | 2.90 |
| | Tube-on-tube 30 cm (300 mL/min) | 0.86 | 666.4 | 1.12 | 1.31 |
| | Serpentine 1.85 m (300 mL/min) | 2.65 | 1134.8 | 11.12 | 4.20 |
| | Plate (300 mL/min) | 2.87 | 1092.2 | 10.49 | 3.65 |
| L. Hein & M. Mortean 2021[B] | AM polymer compact HX | 30.3 | 194.0 | 5.0 | 0.17 |
| L. Song et al. 2010[C] | Polymeric hollow-fiber HX for desalination, brine and water | 150 | 2000 | 19.2 | 0.13 |
| M. Arie et al. 2017[D] | AM via sheet laser fabrication, air to water HX | 4.88 | 120 | 5.4 | 1.11 |
| R.P.P. da Silva et al. 2021[E] | 3D SLM printed | 2480 | 400 | 32.0 | 0.01 |
| H. Moon et al. 2021[F] | 3D SLM printed | 44.6 | 598.4 | 33.3 | 0.75 |
| S. Das et al. 2024[G] | 3D printed | x | 1869.3 | x | 0.502 |

[A]Cost: Stretchlon 800 (Nylon) at \$72.30 per kg; polyethylene at \$73.25 per kg; UHMW polyethylene tape at \$776.83 per kg.

[B]Cost: Nylon 12 powder 10 kg for a total of \$999, volume of the core 72.5 mm x 64 mm x 64 mm and $\rho$ = 1.02 gm/cm$^3$. Performance: U = 194 W/m$^2$K maximum in Figure 10 as provided.

[C]Cost: Membrane cost estimation of \$150 selected via observation of literature, with area of 960.8 cm$^2$. Performance: U = 2000 W/m$^2$K provided for HEPP2 filter in Figure 7a.

[D]Cost: Polyethylene at \$73.25 per kg for 5 HXs worth, 2 sheets each, 10 sheets total and used whole device area of 15.5 cm by 29 cm with thickness of 150 μm. Performance: U = 120 W/m$^2$K provided for water side at 8 g/s.

[E]Cost: \$310 per kg for AISI 316L stainless steel, volume of 100 mm x 100 mm x 100 mm. Performance: U = 400 W/m$^2$K from Figure 15 at max flow rate; gives UA and area of HT.

[F]Cost: Aluminum powder at \$193.95 for 1 kg mass using a mass of 0.23 kg, R = 30 K/kW (and thus U= 33.33 W/K) at Re = 1000 provided in Figure 3.

[G]Cost: Provided cost/UA = 1994 \$/(W/K) in Table 7 (UA/cost = 0.504 W/(\$*K)) in Table 7. Performance: U is estimated through extraction of data from the average U for EOS M290 2% $\Delta$P, $\varepsilon$ = 80% in Figure 16c.

**Table S2.** Expanded comparison of the performance of heat exchangers from **Figure 1.**

| Category | Work | U ($W/m^2K$) | UA/cost (W/K/USD) |
|---|---|---|---|
| This work | 15 cm Tube-on-tube (300 mL/min) | 992.36 | 1.95 |
| | 15 cm Tube-on-tube (500 mL/min) | 1289.46 | 2.53 |
| | 15 cm Tube-on-tube (700 mL/min) | 1479.44 | 2.90 |
| | 1.85 m Tube-on-tube (300 mL/min) | 1134.78 | 4.20 |
| | Plate (300 mL/min) | 1092.20 | 3.65 |
| Metal and polymer heat exchanger works with UA/Cost | L. Song (2010) | 2000.00 | 0.13 |
| | M. Arie (2017) | 120.00 | 1.11 |
| | L. Hein (2021) | 194.00 | 0.17 |
| | R. da Silva (2021) | 400.00 | 0.01 |
| | H. Moon (2021) | 598.44 | 0.75 |
| | S. Das (2024) | 1869.34 | 0.50 |
| Polymer heat exchanger works without UA/Cost | Morcos and Shafey (1995) | 150.00 | x |
| | Liu (2000) | 1100.00 | x |
| | Zarkadas (2004) | 1314.00 | x |
| | Wharry (1999) | 567.00 | x |
| | Bandelier (1992) | 500.00 | x |
| | Astrouski (2013) | 2100.00 | x |

**Section S4: Analytical Modeling and Uncertainty Calculations**

***Tube-on-tube heat exchanger modeling***

We begin with defining a given design with a prescribed geometry of the tube-one-tube heat exchanger of overlap fraction, device length, flat-channel length, square root of cross-sectional area (as a substitute for diameter), and cross-sectional area ($A_{xc}$). Additionally, we define inlet temperatures of the cold (10 °C) and hot flow (60 °C) along with the fluid's physical properties for the density, heat capacity rates, thermal conductivity, kinematic viscosity, and thermal diffusivity evaluated at respective temperatures detailed in **Table S3**.

**Table S3.** Properties of water used at 10 °C and 60 °C

| **Thermophysical Properties of Water** | **Hot (60 °C)** | **Cold (10 °C)** |
|---|---|---|
| Density ($kg{\cdot}m^{-3}$) | 983.2 | 999.7 |
| Heat Capacity Rate ($J{\cdot}kg^{-1}{\cdot}K^{-1}$) | 4190 | 4200 |
| Thermal Conductivity ($W{\cdot}m^{-1}{\cdot}K^{-1}$) | 0.651 | 0.579 |
| Kinematic Viscosity ($m^2{\cdot}s^{-1}$) | 4.74E-07 | 1.31E-06 |
| Thermal Diffusivity ($m^2{\cdot}s^{-1}$) | 1.59E-07 | 1.38E-07 |

Next, we identify a range of flow rates to test from 300 mL/min to 1000 mL/min because our experimental tests will utilize this same range, an operational range that captures flow that can range from laminar to turbulent flow depending on the conditions of the fluid. Specifically, the range of temperatures of the fluids (cold inlet of 10 °C and hot inlet of 60 °C) results in a spread in Reynolds numbers across the transition point from laminar to turbulent flow regimes (i.e., where Re = 2300) due to the dependence of the dynamic viscosity and density terms on fluid temperature. The flow rate at which the transition in flow regime occurs (when Re = 2300) decreases as temperature increases, as shown in **Figure S6**.

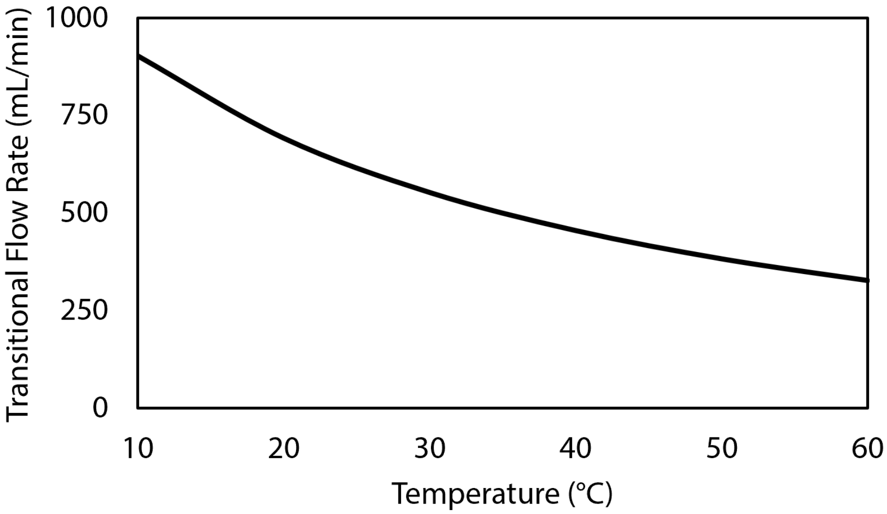


**Figure S6.** Transitional flow rate for the 56% overlap tube-on-tube heat exchanger varying with temperature, corresponding to a transitional Reynolds number of 2300.

The fundamental equations of the prediction process are **Equations S1 and S2**, the energy balance rate of heat transfer in one stream and the overall heat transfer coefficient model. The thermal conductivity of the material for nylon is 0.25 $Wm^{-1}K^{-1}$ and for polyethylene is ~0.40 $Wm^{-1}K^{-1}$. The thickness δ is ~50 μm defined via the thickness of one sheet of the material, which divides the two channels. The remaining unknowns in **Equation S2** are the convection coefficients for each stream.

$$q_{stream} = \dot{m}_{stream} C_{p,stream}(T_{in} - T_{out}) \tag{S1}$$

$$U_{model} = \frac{1}{\sum R} = \frac{1}{\frac{1}{h_{hot}} + \frac{\delta}{k_{mat}} + \frac{1}{h_{cold}}} \tag{S2}$$

We identify these terms in the $U_{\text{model}}$ equation through **Equation S3**, which require calculating the Nusselt number for each fluid stream temperature. The Nusselt number is a dimensionless number that relates convective and conductive heat transfer as defined in **Equation S4** and are used for a variety of flows via experimental results published in the past.

$$h_{stream} = \frac{Nu_{stream} \cdot k_{stream}}{D} \tag{S3}$$

$$\mathrm{Nu}_{\mathrm{stream}} = \frac{\mathrm{hL}}{\mathrm{k}} \quad \text{(S4)}$$

We set out to determine which Nusselt number correlation best fits our study, but the literature is abundant with Nusselt number correlations to select for various heat exchanger scenarios. We begin by looking at the classic Dittus and Boelter correlation as introduced by McAdams, then to the more widespread and accurate Gnielinski correlations for turbulent and transitional flow for compact heat exchangers.[4–10] These equations come with some limitations, however, including in the transition region where Re < 4000, but primarily the assumption of a circular cross-sectional area inside the correlations can result in greater deviations from experimental results.[10,11] Recently, authors have proposed correlations that take into account the variation of diameters and cross sectional areas through the form of a characteristic length $A_{xc}^{1/2}$. This characteristic length selection results in a general that minimizes the impact of cross-sectional shape, ideal for our use case with the tube-on-tube design and use this $A_{xc}^{1/2}$ term in Equation S3 instead of D.

We select from the literature, correlations that correspond to laminar and the transitional to turbulent regime for non-circular cross-sections using the $A_{xc}^{1/2}$ term in place of the diameter. For laminar flow, we select a correlation from Muzychka and Yovanovich (**Equation S5**) that is valid for Reynolds numbers less than 2100.[12–14]

$$Nu_{\sqrt{A_{xc}},L} = \left[\left(\frac{C_4 f(Pr)}{\sqrt{L^*_{th,\sqrt{A_{xc}}}}}\right)^m + \left(\left(C_2 C_3\left(\frac{fRe_{\sqrt{A_{xc}}}}{L^*_{th,\sqrt{A_{xc}}}}\right)^{\frac{1}{3}}\right)^5 + \left(C_1\left(\frac{fRe_{\sqrt{A_{xc}}}}{8\sqrt{\pi}\varepsilon^{\gamma}}\right)\right)^5\right)^{\frac{m}{5}}\right]^{\frac{1}{m}} \quad \text{(S5)}$$

For transitional and turbulent flow, we select a correlation from Sarmiento et al. based on a Gnielinski correlation (**Equation S6**) that captures the flow of Re > 2300.[15–17]

$$Nu_{\sqrt{A_{xc}},T} = \frac{\left(\frac{fRe_{\sqrt{A_{xc}},T}}{2}\right)\left(1-\frac{886}{Re_{\sqrt{A_{xc}}}}\right)Pr}{1+12.7\left(\frac{f}{2}\right)^{\frac{1}{2}}\left(Pr^{\frac{2}{3}}-1\right)}\left(1+\left(\frac{1.12\sqrt{A_{xc}}}{L}\right)\right)^{\frac{2}{3}}\left(\frac{Pr}{Pr_{wall}}\right)^2 \quad \text{(S6)}$$

Both of these correlations demonstrate good agreement to experimental studies for non-circular ducts, relevant to this work. There are potential deviations that could result in our work due to our unique tube-on-tube design that varies from previous shapes the authors validated their model on; however, the accuracy shown with prior works provided confidence to utilize these models.

**Table S4.** Equations and terms used in the heat exchanger analytical modeling to determine the Nusselt number correlations for turbulent and laminar flow.

| Term | Equation/number used |
|---|---|
| $Re_{critical}$ | 1700 |
| $B_{UHF}$ | 425 |
| $mc_{UHF}$ | 16 |
| ε | 0.5 |
| γ (upper bound) | 1/10 |
| $C_{1\_UHF}$ | 3.86 |
| $C_{2\_average}$ | 3/2 |
| $C_{3\_UHF}$ | 0.501 |
| $C_{4\_local}$ | 1 |
| Blending parameter (m) | $m = 2.27 + 1.65Pr^{1/3}$ |
| f(Pr) for UHF | $f(Pr) = 0.886/((1+(1.909Pr_{hot}^{1/6})^{9/2}))^{2/9})$ |
| Thermal length $L^*_{th,\sqrt{A_{xc}}}$ | $L^*_{th,\sqrt{A_{xc}}} = L_{HX}/\sqrt{A_{xc}}/(RePr)$ |
| Hydrodynamic length $L^*_{hy,\sqrt{A_{xc}}}$ | $L^*_{hy,\sqrt{A_{xc}}} = L_{HX}/\sqrt{A_{xc}}/(Re)$ |
| $fRe_{\sqrt{A_{xc}},L}$ | $fRe_{\sqrt{A_{xc}},L} = \left\{\left(\frac{3.44}{L^{*\frac{1}{2}}_{th,\sqrt{A_{xc}}}}\right)^2 + \left(\frac{12}{\varepsilon^{\frac{1}{2}}(1+\varepsilon)\left[1-\left(\frac{192\varepsilon}{\pi^5}\right)\tanh\left(\frac{\pi}{2\varepsilon}\right)\right]}\right)^2\right\}^{\frac{1}{2}}$ |
| $fRe_{\sqrt{A_{xc}},T}$ | $fRe_{\sqrt{A_{xc}},T} = Re_{\sqrt{A_{xc}}}\left[3.6log_{10}\left(\frac{6.115}{Re_{\sqrt{A_{xc}}}}\right)\right]^{-2}$ |
| $Re_{\sqrt{A_{xc}},L\&T}$ | $Re_{\sqrt{A_{xc}},L\&T} = \frac{V\sqrt{A_{xc}}}{\nu}$ |

Once the Nusselt numbers are found, we calculate $U_{model}$ to proceed with our analysis. We then continue with the heat exchanger performance calculation by using the effectiveness-NTU method using **Equations S7, S8, S9, and S10** to determine the rate of heat transfer by calculating NTU and plugging it into **Equation S8** for a parallel flow orientation or **Equation S9** for counter flow orientation to find the effectiveness. We then can take the effectiveness and can find the $Q_{predicted}$ via the effectiveness in **Equation S11.**

$$NTU = \frac{UA}{C_{min}} \tag{S7}$$

$$\varepsilon = \frac{1 - e^{-NTU(1+C_r)}}{(1 + C_r)} \tag{S8}$$

$$\varepsilon = \frac{1 - e^{-NTU(1-C_r)}}{(1 - C_r \cdot e^{-NTU(1-C_r)})} \tag{S9}$$

$$\varepsilon = \frac{q_{predicted}}{q_{max}} \tag{S10}$$

$$q_{predicted} = \varepsilon C_{min}(T_{hot,in} - T_{cold,in}) \tag{S11}$$

We are then able to predict the output temperatures of the hot and cold channels of the heat exchanger to subsequently compare to our results experimentally.

$$T_{hot,out} = T_{hot,in} - \frac{q_{predicted}}{\dot{m}_{hot} c_{p\_hot}} \tag{S12}$$

$$T_{cold,out} = T_{cold,in} + \frac{q_{predicted}}{\dot{m}_{cold} c_{p\_cold}} \tag{S13}$$

**Section S5: Parallel Flow and Counter Flow Models**

For heat exchangers, the flow orientation is one of the most fundamental design decisions underpinning the performance of any system. In this work, we conduct experiments and modeling that capture both flow orientations to motivate the applicability of the sheet-based designs in both parallel flow and in counter flow. In **Figure 2**, the analytical models shown are the parallel flow model only, due to the low (< 0.1%) percentage difference from the counterflow model. Here, we further investigate this small deviation at compact lengths and seek to visualize the growth of the difference of counter flow performance over the parallel flow case as the length of the heat exchangers increases. For all model results in **Section S5**, inlet temperatures remain at 60 °C and 10 °C and the overlap fraction remains at 56% overlap. A depiction of the small difference in performance characteristics of the experiments detailed in the main text are shown graphically in **Figure S7 and S8** for compact lengths.

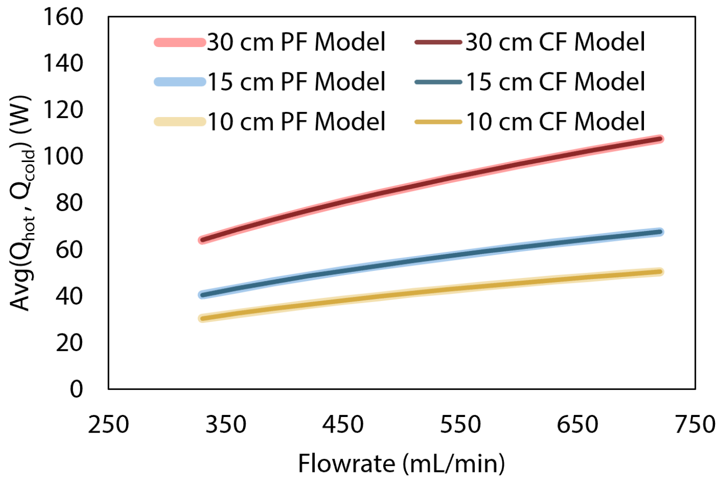


**Figure S7.** Parallel flow (PF) analytical model contrasting with the counter flow (CF) model, demonstrating the closeness between the models at a variety of lengths relevant to the results shown in this work.

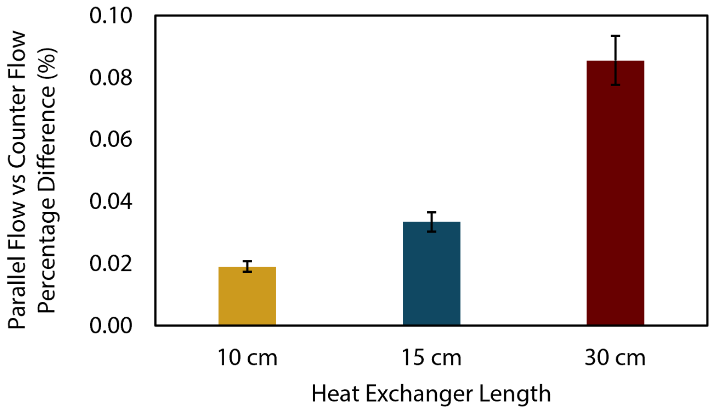


**Figure S8.** Comparison of the percentage difference between parallel flow and counter flow results in terms of Q with a margin of error for a 95% confidence interval.

To visualize the differences in performance between counter flow and parallel flow, we extend the analytical models to larger lengths, beginning with the compact heat exchanger lengths of 10 cm, 15 cm, and 30 cm, then progressing to 2 meters which is representative of our serpentine heat exchanger design, and finally scaling up to one million meters to represent the case where length and area of heat exchange go to infinity, shown in **Figure S9**. We model the difference between counter flow and parallel flow in **Figure S10** for three distinct flow rates. The resulting heat exchange of the counter flow case will become two times greater than the parallel flow case as the heat exchanger length approaches infinity. This 2x increase occurs as the length goes to infinity because the parallel flow streams equilibrate to the same temperature at their outlets, contrasting with the counter flow outlets, which cross over to the opposite stream's input temperature (e.g. the hot stream inlet of 60 °C will become 10 °C) in the case of identical flow rates and fluid specific heats, which allows the counter flow to achieve double the rate of heat transfer.

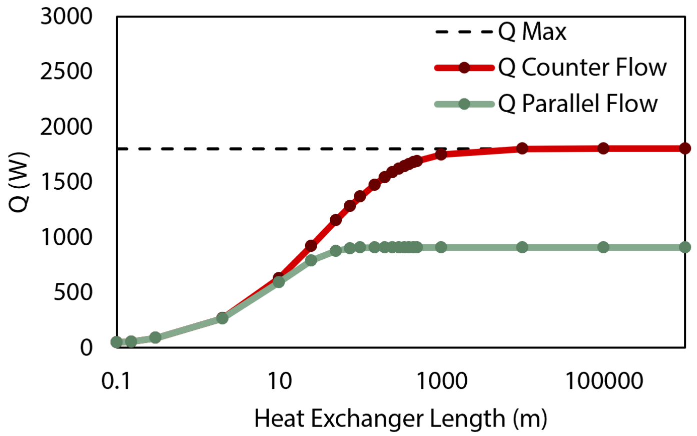


**Figure S9.** Heat transfer rates of counter flow and parallel flow deviating as the length of the heat exchanger increases, with the counter flow approaching the maximum possible rate of heat transfer for a constant effective diameter and area of heat transfer (overlap fraction of 56%).

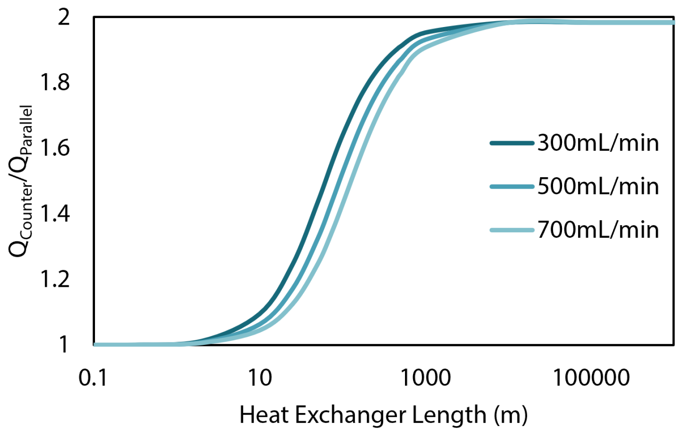


**Figure S10.** Ratio of heat transfer rates of counter flow versus parallel flow heat exchangers with a constant overlap fraction of 56% and constant effective diameter, shown as length goes to infinity for water with the same specific heat characteristics and same flow rates in both streams.

**Section S6: Uncertainty Calculations**

For accounting for the uncertainty in our data measurements, we included the instrumentation error along with the standard deviation for any collected data set that includes temperature, pressure, and flow rate. For temperature, with 1% instrumentation error and for the flow rate at a 3% error along with each standard deviation of 600 data points. For the rate of heat transfer, the error of each term must be incorporated as the error propagates. Additionally, this process flows to calculate the LMTD temperature difference as well. **Equations S14-S17** are involved in subsequent uncertainty calculations, but **Equations S16 and S17** primarily influence the **Equation S18** for the uncertainty of the heat transfer rate.

$$\delta T_{stream,in} = \sqrt{(0.01 \cdot T_{in})^2 + \sigma^2_{T_{in\_std}}} \tag{S14}$$

$$\delta T_{stream,out} = \sqrt{(0.01 \cdot T_{out})^2 + \sigma^2_{T_{out\_std}}} \tag{S15}$$

$$\delta \Delta T_{stream} = \sqrt{(0.01 \cdot \Delta T_{stream})^2 + \sigma^2_{\Delta T_{stream}}} \tag{S16}$$

$$\delta \dot{m}_{stream} = \sqrt{(0.03 \cdot \dot{m}_{stream})^2 + \sigma^2_{\dot{m}_{stream\_std}}} \tag{S17}$$

$$\delta q_{stream} = q_{stream} \cdot \sqrt{\left(\frac{\delta \dot{m}_{stream}}{\dot{m}_{stream,avg}}\right)^2 + \left(\frac{\delta \Delta T_{stream}}{\Delta T_{stream}}\right)^2} \tag{S18}$$

The pressure sensor additionally has uncertainty associated with the instrumentation at 1%, and is detailed in **Equations S19-S21**.

$$\delta P_{in} = \sqrt{(0.01 \cdot P_{in})^2 + \sigma^2_{P_{in}}} \tag{S19}$$

$$\delta P_{out} = \sqrt{(0.01 \cdot P_{out})^2 + \sigma^2_{P_{out}}} \tag{S20}$$

$$\delta\Delta P_{total} = \sqrt{(\delta P_{out})^2 + (\delta P_{in})^2} \tag{S21}$$

**Equation S22** calculates the uncertainty of the log mean temperature difference where the change in temperature varies whether or not the flow is parallel (PF) or counter flow (CF). The subsequent terms that make up **Equation S22** are in **Equation S23-S26.**

$$\delta\Delta T_{LMTD} = \sqrt{\left[\left(\frac{1}{\ln\left(\frac{\Delta T_1}{\Delta T_2}\right)} - \frac{\Delta T_1 - \Delta T_2}{\Delta T_1\left(\ln\left(\frac{\Delta T_1}{\Delta T_2}\right)\right)^2}\right)\delta\Delta T_1\right]^2 + \left[\left(-\frac{1}{\ln\left(\frac{\Delta T_1}{\Delta T_2}\right)} + \frac{\Delta T_1 - \Delta T_2}{\Delta T_2\left(\ln\left(\frac{\Delta T_1}{\Delta T_2}\right)\right)^2}\right)\delta\Delta T_2\right]^2} \tag{S22}$$

Where:

$$\delta\Delta T_{1,PF} = \sqrt{(\delta T_{hot,in})^2 + (\delta T_{cold,in})^2} \tag{S23}$$

$$\delta\Delta T_{2,PF} = \sqrt{(\delta T_{hot,out})^2 + (\delta T_{cold,out})^2} \tag{S24}$$

$$\delta\Delta T_{1,CF} = \sqrt{(\delta T_{hot,in})^2 + (\delta T_{cold,out})^2} \tag{S25}$$

$$\delta\Delta T_{2,CF} = \sqrt{(\delta T_{hot,out})^2 + (\delta T_{cold,in})^2} \tag{S26}$$

Lastly, incorporating the uncertainty of the rate of heat transfer and the log mean temperature difference, we determine the uncertainty of the overall heat transfer coefficient in **Equation S27**.

$$\delta U_{LMTD} = \mathrm{U}\sqrt{\left(\frac{\delta\Delta T_{LMTD}}{\Delta T_{LMTD}}\right)^2 + \left(\frac{\delta Q}{Q}\right)^2} \tag{S27}$$

To determine which sources of uncertainty are dominant, we can conduct a sensitivity analysis. For example, a 30 cm tube-on-tube heat exchanger in a parallel flow regime at 300 mL/min has an overall uncertainty of 22% for Q and 22% for U. Contributing to the overall uncertainty for both Q and U are the $\delta T$ and $\delta m_{dot}$ uncertainties which define the instrumentation uncertainty in this work. Setting the uncertainty of the $\delta T$ term to zero while keeping the $\delta m_{dot}$ term unchanged yields Q and U uncertainties of 6% each. On the other hand, setting the uncertainty of the $\delta m_{dot}$ term to zero while keeping the $\delta T$ term unchanged yields Q and U uncertainties of 21% each; therefore, the $\delta T$ term uncertainty dominates.

**Section S7: Thermal Conductance Analysis**

The dominance of the convective heat transfer resistance in sheet-based polymer heat exchangers enables high-level performance even when made from low thermal conductivity materials. As **Figure 3** in the main text demonstrates, when the conductive resistance of the polymer sheet separating the fluid streams increases by 2x, the performance of the heat exchanger reduces by only 22%. We can generalize this approach through first defining the sum of resistances for the thermal network in **Equation S28** for a sheet thickness of δ and **Equation S29** for the case of 2δ.

$$R_{total,\delta} = 1/(UA)_{\delta} = R_{convection,hot} + R_{conduction} + R_{convection,cold} \quad (S28)$$

$$R_{total,2\delta} = 1/(UA)_{2\delta} = R_{convection,hot} + 2 \cdot R_{conduction} + R_{convection,cold} \quad (S29)$$

After defining the resistances, we can combine the convection terms via **Equation S30** to simplify the expressions and define the ratio of the two total resistances in **Equation S31**.

$$R_{convection,total} = R_{convection,hot} + R_{convection,cold} \quad (S30)$$

$$\frac{R_{total,2\delta}}{R_{total,\delta}} = \frac{2 \cdot R_{conduction} + R_{convection,total}}{R_{conduction} + R_{convection,total}} \quad (S31)$$

We can use **Equation S31** and define the ratio of total resistances via our experimental data and plug in that value to isolate for the relationship between $R_{conduction}$ and $R_{convection}$ to determine what percentage of the total resistance is due to conduction.

In prior work, the polymer material from which a heat exchanger is constructed has sometimes been seen as a limitation due to its low thermal conductivity. For our case in which doubling the polymer thickness resulted in only a 22% decrease in performance, yielding a resistance ratio from **Equation S31** of 1.22 to apply in the following equations. Plugging in this value, we can rearrange to **Equation S32** through multiplying out the denominator. Simplifying via **Equation S33** results in a conduction and convection relationship that designates the convection term as larger in **Equation S34**.

$$1.22 \cdot R_{conduction} + 1.22 \cdot R_{convection,total} = 2 \cdot R_{conduction} + R_{convection,total} \tag{S32}$$

$$0.78 \cdot R_{conduction} = 0.22 \cdot R_{convection,total} \tag{S33}$$

$$R_{conduction} = 0.28 \cdot R_{convection,total} \tag{S34}$$

As shown in **Equation S34**, the convective terms make up ~80% of the total resistance when comparing to the result that includes conduction in **Equation S28**. This ratio demonstrates that even when the thermal conductivity goes to infinity as defined in **Equations S35 and S36**, and the conduction thermal resistance correspondingly goes to zero, the performance for these given inputs will remain constrained by the convective terms.

$$R_{conduction} = \frac{\delta}{kA} \tag{S35}$$

$$R_{k\to\infty} = 1/(UA)_{k\to\infty} = R_{convection,total} \tag{S36}$$

Specifically, for $R_{k\to\infty}$, it can be calculated that $(UA)_{k\to\infty} = 1.28 \cdot (UA)_{\delta}$, which shows that the maximum performance even for a perfectly thermally conductive material would only be 28% greater than the performance shown in this work based on a fully polymer construction.

**Section S8: Deployability**

The deployability in **Figure 1g** is based on the initially thin state of the heat exchanger achieved via the sheet-based architecture. For the deployability, we compare the volume of each device in its uninflated state, similar to a flat sheet, to its inflated volume. We fill each device with the maximum amount of liquid it can hold while the exit ports are fully blocked to prevent the liquid from traveling through the heat exchanger. We take the ratio between the fully inflated volume and the uninflated volume to calculate our deployability ratio. Further detailed in **Figure S11**, we show the state of the heat exchanger in an undeployed and a deployed state. Furthermore, we describe schematically how the volume of the plate heat exchanger increases by 60 times in **Figure S12**, where we show that the inflation of the device constructed with tufts (small bonded circular regions) allows for a high level of deployability.

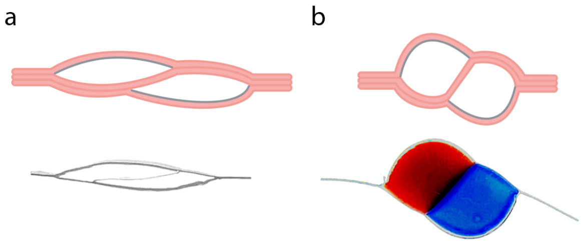


**Figure S11.** Cross-sectional images of the tube-on-tube architecture in an (a) undeployed state and in an (b) active inflated state. The lower image in (b) was obtained by injecting pressurized uncured liquid elastomer into each flow channel of the heat exchanger, with red and blue elastomer dye used to visually set apart the hot and cold streams, and then allowing the elastomer to cure before physically cross-sectioning and imaging the slice of the heat exchanger shown here with backlighting for visualization.

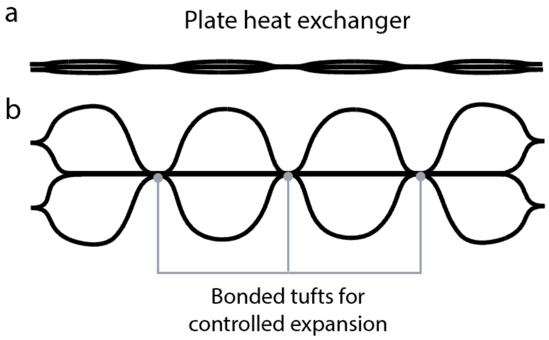


**Figure S12.** The cross-sectional view of the plate heat exchanger, in which the channels are formed via the introduction of tufts (small bonded circular regions) to control the expansion of the heat

exchanger. We compare the (a) initial volume to the (b) deployed volume to validate the 60 times volume increase when in use.

**Section S9: Pressure Limitation**

The pressure limits of a heat exchanger must be characterized to ensure that the heat exchanger operates effectively and without risk of failure. For our polymeric sheet-based heat exchangers, the expected operational pressures are defined by our pump curve in **Figure S2** that shows a maximum pressure head of five meters, or 50 kPa. Therefore, in our design process, we selected sheets of thermoplastics including nylon and polyethylene that could withstand this requirement.

In addition to ensuring safe operation with our pumps, we also characterized the maximum pressure for the tube-on-tube heat exchangers shown in this work to determine the operational limitations of these devices for general use. In prior work, we developed a model for burst failure of sheet-based devices that we applied to our sheet-based heat exchangers to predict the maximum pressure at which they can operate.[18] To inform this model, we first conducted T-peel tests, where we bonded a selected region of two thermoplastic sheets together and subsequently placed the free ends of each sheet (the "T-peel arms")[18,19] into a universal testing machine (Instron, 68SC-2) where we conducted tensile tests on the bonded sheets to obtain their force-displacement curves. After conducting 5 trials, we averaged the results to obtain the bond strength for the sheets and input this strength (in the form of an averaged force-displacement curve, **Figure S13a**) into our model to determine the pressure versus both the characteristic length and the tensile force within the sheets (detailed in the diagram in **Figure S13b**), shown in **Figure S13c** and **Figure S13d**. The maximum pressure according to the model is indicated by the peak and is approximately 200 kPa for our system. The pressures and lengths/forces to the left of each peak are stable; the values to the right are unstable in a pressure-controlled system and would result in runaway expansion and bursting, which is why the peak pressure in each plot is identical to the burst pressure. It is also important to note from **Figure S13c** that the channels stretch slightly at increased pressures, even in the stable regime before bursting; this modeling approach can be used to determine an operating pressure that limits strain to a specific amount: e.g., to maintain strain < 15%, the operating pressure would be limited to 104 kPa.

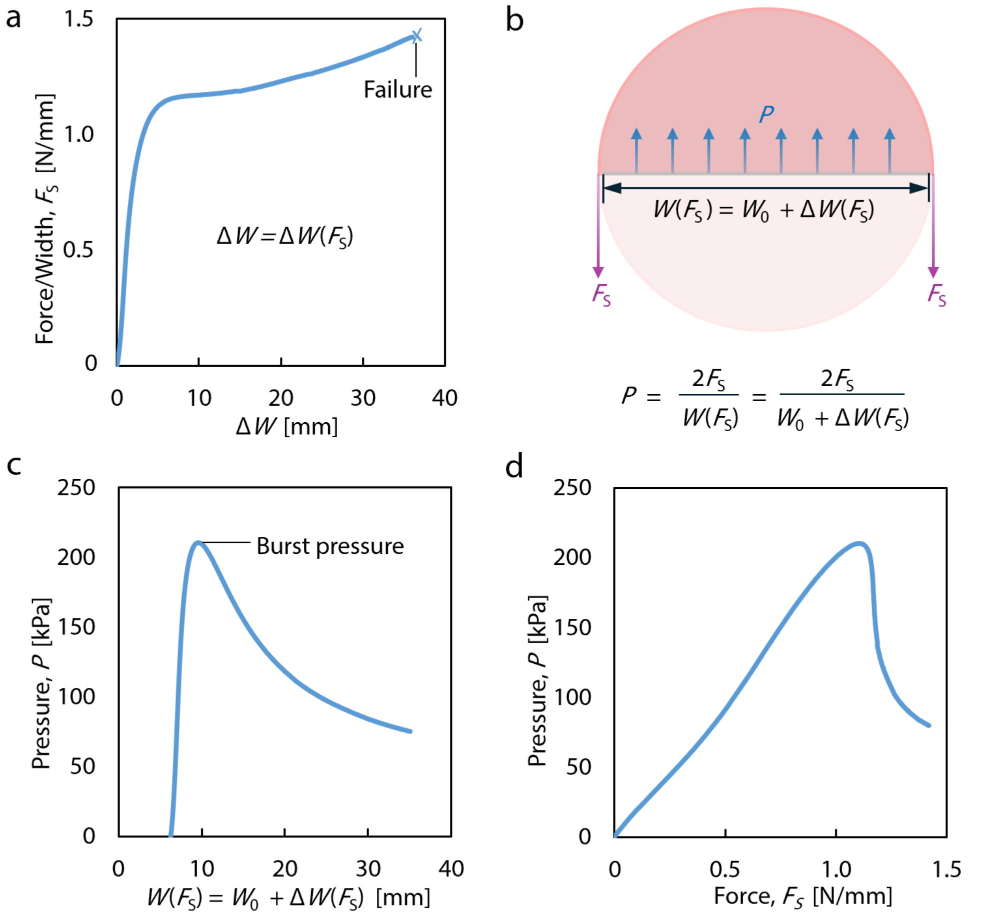


**Figure S13.** Modeling framework and results. (a) T-peel tensile testing data for bonded nylon sheets are converted to expected burst pressure via the modeling framework shown schematically in (b). Burst pressure model results showing (c) pressure versus the characteristic length, W, of the channel for fluid flow and (d) pressure versus the tensile force within the bonded sheets.

We then conducted burst tests of our sheet-based heat exchangers, with results shown in **Figure S14**. We connected each heat exchanger to an electronically regulated (McMaster Carr, 8083T2) compressed air line instrumented with a pressure sensor (Panasonic, ADP5171); both fluid streams of each heat exchanger were attached in parallel to the same pressure source. We first tested the tube-on-tube heat exchanger design with a single outer layer, with experimentally determined burst pressures shown for three trials in **Figure S14a**; the sharp drop-off at the end of the region of constant upward slope indicates a bursting event. The experimental results show that the burst pressure values range from ~100–300 kPa with an average value of 180 kPa based on three trials (**Figure S14c**), generally in agreement with our model prediction of 200 kPa but also indicating

the need for a safety factor in device design to account for uncertainty related to the manufacturing process.

We also show that we can further tailor the maximum pressure by increasing the number of layers of the heat exchanger (**Figure S14b-c**), where adding a second exterior layer increases the burst pressure by approximately 2x without sacrificing thermal performance (i.e., there is still only a single layer of polymer sheet separating the two fluid streams). We conclude that, for higher pressure applications, increasing the number of bonded external layers can increase the maximum burst pressure and thus results in a higher operating pressure. Additionally, the added external layers will provide greater insulation from the environment, resulting in reduced heat exchange with the environment as a secondary benefit. Future work could involve further investigation into the limitations of the maximum pressures that are possible for different materials and for an increasing number of layers, or for differential pressures across the two fluid streams.

To explore the influence of elevated temperature on operational limits, **Figure S14d** shows the burst pressure results for the same device geometry as in **Figure S14a**, but at an elevated temperature of 70 °C, which is greater than all the temperatures encountered in our work (i.e., the maximum temperature was 60 °C for the hot stream) to ensure that the epoxy and connection ports remain intact during operation. The results in **Figure 14d** indicate that this higher environmental temperature does not compromise performance below the 50 kPa maximum supplied from our pumps. The failure temperature will vary depending on material selection and bonding method for thermoplastic materials; this temperature is an important design parameter for future work on heat exchangers that are resistant to higher temperatures. For the two thermoplastics discussed in this work, nylon (Stretchlon 800, Fibre Glast) and polyethylene (Polyethylene Bagging Film, Fibre Glast), the maximum operational temperatures are 205 °C and 82 °C, respectively.[20,21]

We also quantified the hermeticity of the epoxy and ports at elevated temperatures by placing the heat exchanger into a temperature-controlled chamber with both of the heat exchanger channels connected in parallel to an air pressure source and pressure sensor (Honeywell, ABP2MANG001BGAA5XX). Once the heat exchanger is placed inside the chamber, the chamber heats up to the set temperature and, upon reaching it, we then close a valve isolating the heat exchanger flow channels and disconnect the input pressure supply to ensure that there could be no source of additional pressurized air. The heat exchanger maintained a constant internal pressure of

~7 kPa at 70 °C in **Figure S15** along with a corresponding mass loss rate of approximately zero as shown in **Figure S16** over a greater period of time than a typical heat exchanger test (1000 seconds versus 600 seconds).

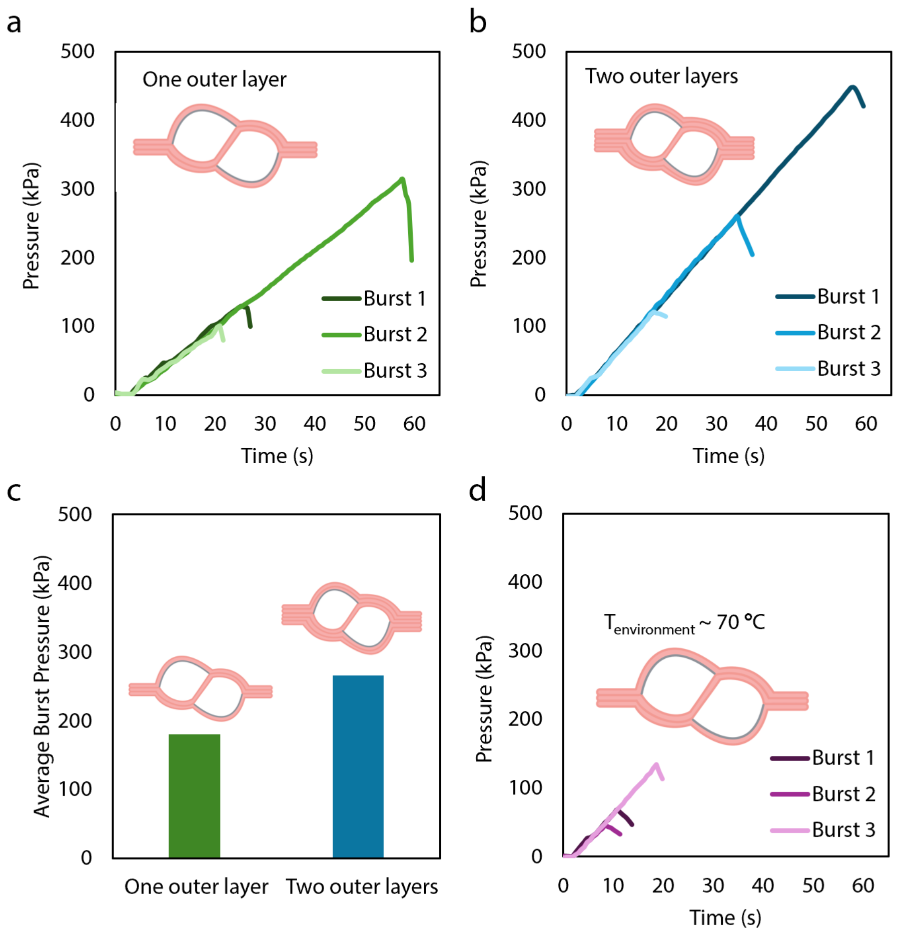


**Figure S14.** Burst pressure testing. (a) Burst test at ambient temperature for a tube-on-tube heat exchanger with a single outer layer, like the majority of the heat exchangers presented in the main text. (b) Burst test at ambient temperature for a tube-on-tube heat exchanger with a double outer layer. (c) Comparison of average burst pressure between the single layer and double layer, showing that adding a second exterior layer increases the burst pressure by approximately 2x without sacrificing thermal performance because there is only one polymer sheet separating the working fluids. (d) Burst test in an elevated temperature environment, higher than the maximum temperature case that we encountered in this work (60 °C), maintaining a hermetic seal at the higher temperature until reaching burst pressure, which was lower than the burst pressure at room temperature.

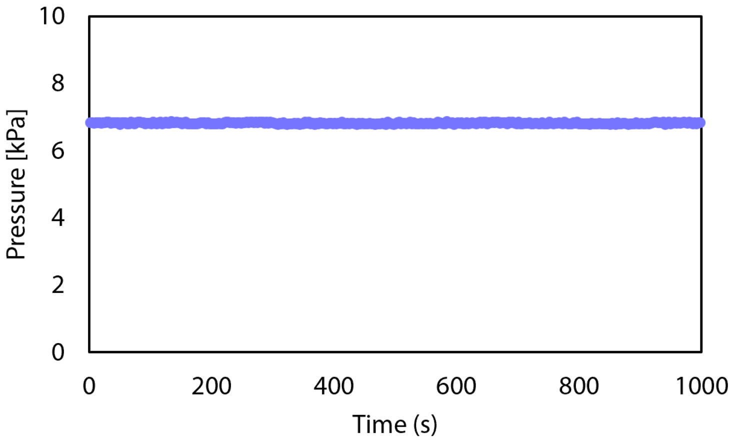


**Figure S15.** Sheet-based heat exchanger maintenance of pressure at 70 °C, ensuring the function of the port attachments and epoxy in elevated temperature environments.

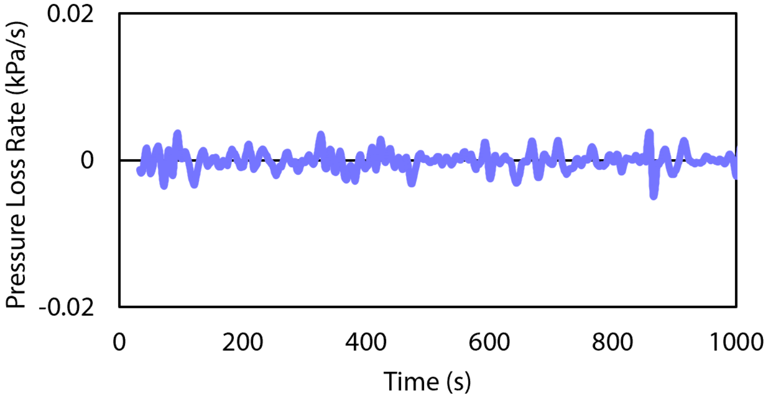


**Figure S16.** Rate of pressure loss of the sheet-based heat exchanger in the 70 °C temperature environment; the rate is approximately 0 kPa/s over the course of 1000 s. The fluctuations in data are attributed to the reported instrument uncertainty in recording the pressure loss rate of ±0.025 kPa/s.

**Other Supporting Information for This Manuscript Includes the Following:**

**Movie S1: Flow visualization.** Demonstration of injecting dyed fluid to visualize laminar and turbulent fluid flow. A needle was manually inserted at the inlet side of one channel of the heat exchanger nearly parallel to the flow direction for a representative flow visualization.

**Movie S2: Acid resistance of tube-on-tube polymeric heat exchanger.** Comparison of the performance of different thermoplastic materials demonstrating the capabilities of select thermoplastics to handle corrosive (acidic) environments.

**Supporting Information References**


[1] F. Connolly, D. A. Wagner, C. J. Walsh, K. Bertoldi, *Extreme Mechanics Letters* **2019**, *27*, 52.
[2] H. Moon, D. J. McGregor, N. Miljkovic, W. P. King, *Joule* **2021**, *5*, 3045.
[3] L. L. Hein, M. V. V. Mortean, *International Communications in Heat and Mass Transfer* **2021**, *124*, 105237.
[4] F. W. Dittus, *Univ. of California Pub., Eng.* **1930**, *2*, 443.
[5] W. H. McAdams, *Heat Transmission*, 2nd Edition., McGraw-Hill **1942**.
[6] E. N. Sieder, G. E. Tate, *Ind. Eng. Chem.* **1936**, *28*, 1429.
[7] R. H. S. Winterton, *International Journal of Heat and Mass Transfer* **1998**, *41*, 809.
[8] V. Gnielinski, *International Chemical Engineering* **1976**, *16*, 359.
[9] V. Gnielinski, *Forschung im Ingenieurwesen* **1995**, *61*, 240.
[10] P. Stephan, Ed., *VDI Heat Atlas*, 2nd Edition., Springer Berlin Heidelberg, Berlin, Heidelberg **2010**.
[11] R. K. Shah, A. L. London, *Defense Technical Information Center* **1971**.
[12] Y. Muzychka, M. Yovanovich, *2nd AIAA, Theoretical Fluid Mechanics Meeting* **1998**, 2492.
[13] Y. Muzychka, M. Yovanovich, *7th AIAA/ASME Joint Thermophysics and Heat Transfer Conference* **1998**, 2586.
[14] Y. S. Muzychka, M. M. Yovanovich, *Journal of Heat Transfer* **2004**, *126*, 54.
[15] A. P. C. Sarmiento, V. H. T. Soares, F. H. Milanez, M. B. H. Mantelli, *International Journal of Heat and Mass Transfer* **2020**, *149*, 119165.
[16] A. P. C. Sarmiento, V. H. T. Soares, G. G. Carqueja, J. V. C. Batista, F. H. Milanese, M. B. H. Mantelli, *International Journal of Thermal Sciences* **2020**, *153*, 106384.
[17] A. P. C. Sarmiento, F. H. Milanez, M. B. H. Mantelli, *Applied Thermal Engineering* **2021**, *184*, 115435.
[18] A. Broshkevitch, S. Urbina, B. Jumet, J. A. Garavito-Leon, A. Rajappan, D. J. Preston, *Cell Reports Physical Science* **2025**, *6*, 102437.
[19] T. F. Yap, J. Klinkao, S. Urbina, N. T. Pottackal, M. D. Bell, A. Rajappan, D. Yavas, D. J. Preston, *Sci. Adv.* **2025**, *11*, eadv2681.
[20] Fibre Glast Polyethylene Bagging Film Product Data Sheet.
[21] Fibre Glast Stretchlon 800 Bagging Film Product Data Sheet.
